# Strategies for combating bacterial biofilms: A focus on anti-biofilm agents and their mechanisms of action

**DOI:** 10.1080/21505594.2017.1313372

**Published:** 2017-03-31

**Authors:** Ranita Roy, Monalisa Tiwari, Gianfranco Donelli, Vishvanath Tiwari

**Affiliations:** a Department of Biochemistry, Central University of Rajasthan, Ajmer, India; b Microbial Biofilm Laboratory, IRCCS Fondazione Santa Lucia, Rome, Italy

**Keywords:** anti-biofilm molecules, antimicrobial peptide, biofilm formation, biofilm model, drug resistance, herbal molecules

## Abstract

Biofilm refers to the complex, sessile communities of microbes found either attached to a surface or buried firmly in an extracellular matrix as aggregates. The biofilm matrix surrounding bacteria makes them tolerant to harsh conditions and resistant to antibacterial treatments. Moreover, the biofilms are responsible for causing a broad range of chronic diseases and due to the emergence of antibiotic resistance in bacteria it has really become difficult to treat them with efficacy. Furthermore, the antibiotics available till date are ineffective for treating these biofilm related infections due to their higher values of minimum inhibitory concentration (MIC) and minimum bactericidal concentration (MBC), which may result in *in-vivo* toxicity. Hence, it is critically important to design or screen anti-biofilm molecules that can effectively minimize and eradicate biofilm related infections. In the present article, we have highlighted the mechanism of biofilm formation with reference to different models and various methods used for biofilm detection. A major focus has been put on various anti-biofilm molecules discovered or tested till date which may include herbal active compounds, chelating agents, peptide antibiotics, lantibiotics and synthetic chemical compounds along with their structures, mechanism of action and their respective MICs, MBCs, minimum biofilm inhibitory concentrations (MBICs) as well as the half maximal inhibitory concentration (IC_50_) values available in the literature so far. Different mode of action of anti biofilm molecules addressed here are inhibition via interference in the quorum sensing pathways, adhesion mechanism, disruption of extracellular DNA, protein, lipopolysaccharides, exopolysaccharides and secondary messengers involved in various signaling pathways. From this study, we conclude that the molecules considered here might be used to treat biofilm-associated infections after significant structural modifications, thereby investigating its effective delivery in the host. It should also be ensured that minimum effective concentration of these molecules must be capable of eradicating biofilm infections with maximum potency without posing any adverse side effects on the host.

## Introduction

Biofilm refers to the complex communities of microbes that may be found attached to a surface or may form aggregates without adhering to a surface, as seen in *Pseudomonas aeruginosa, Staphylococcus aureus*, and some other bacteria^1-3^ and buried firmly in an extracellular matrix (ECM). The biofilm lifestyle allows the bacteria to withstand hostile environmental conditions like starvation, desiccation and makes them capable to cause a broad range of chronic diseases. Hence, it is considered as a major cause of persistent nosocomial infections in immune-compromised patients.^4,5^ Around 50% of the nosocomial infections are confined to the patients by indwelling devices used for the purpose of medical treatments such as catheters, cardiac pacemakers, joint prosthesis, dentures, prosthetic heart valves and contact lenses.^6,7^ These foreign bodies provide an ideal surface for the attachment of bacterial cells. Thus a significant increase in biofilm formation has been observed in the presence of implants.^8^ In many cases, the use of antibiotics like imipenem, colistin and many more can only reduce the biofilms but cannot eliminate the entire biofilm. Due to their toxic and side effects it is not possible to reach the minimal concentration of antibiotic *in-vivo*. The higher values of MIC and MBC for the biofilm bacterial cells have therefore made the antibiotic treatment less adequate.^6,9-11^

Moreover, biofilms protect the invading bacteria against the immune system of host via impaired activation of phagocytes and complement system^12-14^ and also increase their resistance against the conventional antibiotics by around 1000-fold.^2,6,15-20^ Some other factors may also account for this antimicrobial tolerance. Previous experimental works revealed several reasons behind the resistance, which includes nature and structure of biofilm, nutrient and oxygen availability to the bacterial cells and intrinsic and acquired bacterial resistance. The involvement of biofilm in providing resistance was made evident from a study on *P. aeruginosa* where the mucoid nature of biofilm was found responsible for high resistance toward tobramycin.^21^ The metabolic state of biofilm-associated bacteria is another potential reason of antimicrobial resistance. Cells of the nutrient depleted zones (slow growing state) in the biofilm may lead to dormancy like the stationary phase which makes the bacteria insensitive to antibiotics since they divide very infrequently.^22,23^ Dividing cells are sensitive to some antibiotics including β lactams, thereafter making them unfit for use. Walters et al. reported that antibiotic resistance was also influenced by limited oxygen supply as observed in case of *P. aeruginosa*, where antibiotic was effective at the air-biofilm interface, the part of the biofilm exposed to oxygen (50–90 μm in the biofilm).^24^ Moreover, studies also demonstrate that biofilm cells undergo a higher rate of mutation than their planktonic counterparts resulting in a 10-fold increase in the efficiency of transfer of plasmid having antibiotic resistance gene, when biofilm is exposed to a sub-lethal concentration of that antibiotic.^25^

Bjarnsholt et al. demonstrated that mucoid biofilms observed in the samples of cystic fibrosis lungs were not found adhered to the lung epithelia, instead were adhered to the neighboring bacteria embedded in a biopolymeric matrix known as aggregated form.^2^ These findings were subsequently confirmed in the studies performed by other researchers^1,3^ suggesting that non-surface attached aggregates exhibit similar levels of tolerance to various antibiotics and polymorphonuclear leukocytes (PMN) as surface attached biofilms. The properties of non-surface attached aggregates despite being similar to that of biofilms, exhibit some differences like they have higher metabolic activity than cells in biofilm as well as planktonic cells. These aggregates have also been reported to be involved in chronic infections and wounds^26,27^ and middle ear infections.^28^ So, the necessity to find new effective drugs that could disperse and eliminate the biofilm is of major concern to prevent and treat various infections caused by generation of biofilms.

Therefore, the study of biofilm and the strategies to eliminate them is one of the most important fields of research in the present days. Many reviews on anti-biofilm compounds have already been done, but this review focuses especially on different strategies or targets of biofilm inhibition. A recent review by Wu et al. has discussed few strategies to combat biofilm. These strategies include the removal of infected foreign bodies like stents and implants and replacing them with new uninfected ones, inhibition of the quorum sensing pathway and modification of c-di-GMP to reduce biofilm infections.^6^ Here, we have made an effort to compile all the known strategies or targets for combating biofilm, which may help the researchers to design new molecules having anti-biofilm activity. Here, we have also discussed the mechanism of biofilm formation with respect to different models followed by various biofilm detection methods along with detailed discussion of the mechanism of action of anti-biofilm molecules found till date. Many of the compounds that exhibit anti-biofilm activity need to undergo further modifications and *in-vivo* tests followed by clinical trials before using them commercially.

## Biofilm formation

Biofilm formation on any surface involves mainly 3 stages. The first stage involves attachment of cells to a surface followed by assembly of the cells to form microcolonies and finally differentiation of biofilm into a mature structure. After the complete development of biofilm, its disassembly or dispersion takes place through both mechanical and active processes.^29^ Deposition of bacteria is especially mediated by sedimentation, Brownian motion and hydrodynamic forces, whereas adhesion to the substratum is governed by Lifshitz–Van der Waals, acid–base, hydrophobic, electrostatic interaction forces.^30^ Certain surface associated proteins like OmpA, fibronectin binding proteins,^31^ protein A,^32^ SasG,^33,34^ biofilm associated protein (BAP)^35,36^ and many other factors are involved in the formation of biofilms, particularly, during initial attachment stages. Some species cannot attach to a surface but can anchor themselves to the matrix or directly to the earlier colonies. Small signaling molecules with the help of cell-cell communication systems mediate this colonization. This phenomenon is generally referred to as quorum sensing.^37^ Biofilm formation is a major quorum-sensing controlled phenotype.^38^ In biofilms, the bacterial cells are enclosed in an extracellular matrix, which is a complex and highly polar mixture of biomolecules including proteins, polysaccharides, nucleic acids and lipids.^39^ The matrix provides protection from various stress conditions such as antimicrobial exposure or immune cells attack. However, the matrix of the biofilm does not act as a mechanical barrier for the antimicrobial agent.^40^ This was confirmed by a study which shows that biofilm formed by β-lactamase-deficient strain of *K. pneumoniae*, allowed the penetration of ampicillin whereas in wild type *K. pneumoniae* strain possessing β-lactamase, ampicillin was unable to infiltrate biofilm,^40^ suggesting that in the latter case, ampicillin was rapidly degraded by β-lactamase before infiltrating the wild type biofilm. Once the bacteria start secreting extracellular polysaccharide substance (EPS), second stage of development of biofilm comes in process, which is an irreversible process. The secretion of EPS is continuous till the third stage of formation ensuring the safe attachment of bacteria to the surface inside a thickly complex bio-molecular layer.^41^ The fully matured biofilm now takes on a tower-like structure having 3 dimensions. These towers comprise of small channels, which transport nutrients, water and waste, and the small cavities present in the towers provide shelter for the planktonic bacteria. Studies also demonstrate that the organization and architecture of biofilms vary greatly for different bacteria. Exact reason for this variation remains unclear. However, the adhesive protein LapA governs the biofilm formation of *P. putida*^42-44^ while exopolysaccharides Pel and Psl govern biofilm formation in other pseudomonads including *P. aeruginosa*.^45-47^ Hence, difference in extracellular matrix (ECM) component may give rise to the variations in the structure of biofilm. Finally, these towers either erode (small parts) or are sloughed off (large parts) and get detached, emptying the cavities containing non-surface attached bacteria. This is followed by the release of fresh bacteria into the environment.^48,49^

Some recent studies on various bacterial species such as *Pseudomonas aeruginosa, Pseudomonas putida, Pseudomonas fluorescens, Yersinia pestis, Escherichia coli, Vibrio cholerae, Burkholderia cenocepacia, Salmonella enterica, Clostridium difficle*, *Klebsiella pneumoniae, Vibrio cholerae* and *Bacillus subtilis* demostrate that increase in c-di-GMP level, an intracellular secondary messenger designates the initiation of biofilm formation and virulence.^42,43,50-58,59-63^ c-di-GMP was first described as a novel secondary messenger in the allosteric activation of cellulose synthase of *Gluconacetobacter xylinus*.^55^ Several types of c-di-GMP diguanylate cyclase and phosphodiesterases that are synthesized by bacteria participate in different c-di-GMP circuits.^64^ c-di-GMP functions by binding to a wide range of receptors which include enzymes, adaptor proteins, transcription factors and riboswitches.^61^ It has also been reported that various environment causes and transducer mechanisms lead to an increase in the c-di-GMP level in the cell. This not only leads to the production of adhesins but also helps in the secretion of extracellular matrix.^65,66^ In *P. aeruginosa*, the level of c-di-GMP positively regulates the production of extracellular matrix components such as CdrA adhesin, alginate exo-polysaccharide, Pel and Psl.^53,67^ Along with c-di-GMP, small regulatory RNAs (sRNA) also regulate the formation of biofilm in several bacterial species.^68^

Certain bacterial strains have the ability to form planktonic aggregates, which depend on growth conditions. Previous studies suggest that some strains of *S. aureus* form large aggregates and the formation process starts in the early exponential growth phase. A cluster of about 20 cells form a structured population when cell density is low. However, at higher density these structures are larger forming aggregates up to diameter of 1000µm. Extracellular polysaccharide intracellular adhesin (referred as polymers of β 1–6 N-acetylglucosamine or PNAG after determination of the chemical structure),^69^ and *spa* encoding Protein A are reported to be responsible for the extensive aggregation.^3^ Studies by Alhede et al. 2011 suggested that the matrix of aggregates of *P. aeruginosa* comprises of DNA and mannose-rich extracellular polysaccharide like Psl.^1^

## Biofilm models

Study of various biofilm model systems enhances the knowledge regarding the biofilm biology. The biofilms are studied using both *in-vivo* and *in-vitro* model systems. *In-vitro* biofilm model systems are broadly classified into 3 major types including closed or static model, open or dynamic models and microcosms. The most frequently used closed model systems are microtitre plate-based model systems which uses static and batch growth conditions.^70^ In this model, there is no flow of media, product or waste materials into or out of the reactor, so the experimental conditions changes gradually in the wells like accumulation of signaling components, increase of bacterial population and depletion of nutrients in media. Since, it is cost effective and require small volume of reagents therefore, numerous tests can be performed at a single time.^71^ Additionally, microtitre plate-based models can be used to differentiate between biofilm-deficient mutants and biofilm forming wild type strains,^72,73^ determine the antimicrobial and anti-biofilm effects of different antimicrobial compounds, identify the factors involved in the biofilm initiation such as adhesins, pili, flagella, enzymes involved in cyclic-di-GMP metabolism and genes responsible for extracellular polysaccharide production.^74,75^ Among the open and dynamic models, flow displacement biofilm model is most commonly used to study biofilms. Unlike the microtitre plate method, in this model system, addition of nutrients and release of waste products can occur.^70,76^ The dynamic model of biofilm formation using perfused silicone tubes is one of the most important models for studying biofilms as it initiates *in-vivo* conditions very closely. Biofilms are formed in a silicone tube system under dynamic condition followed by cutting of the tube in small pieces for further treatment and investigation.^77^ Microcosms constitute another *in-vitro* model system for studying biofilms that mimic with the *in situ* conditions in controlled environment, such as for studying wound biofilm, oral biofilm, stream biofilm and dental biofilm.^78-80^ Both *in-vitro* and *in-vivo* systems can be turned into a microcosm by using the same medium and creating an artificial environment to assess the cell metabolism and behavior. Apart from this there exists an *ex-vivo* model system, which deals with the tissues and organs extracted from organisms for the further analysis and experimentation in artificial environment. This model can be useful to monitor the bacterial colonization and progression in the given tissue or organ. To validate the simplified results provided by the *in-vitro* model studies, certain *in-vivo* model system studies should be performed. To address various therapeutic and diagnostic challenges the studies of mammalian models closer to the humans is necessary. These tissue-associated model systems are being used for studying mainly lung infections, urinary tract infections as well as the wound infections.^74,81^ Different other models such as central venous catheter models; subcutaneous foreign body infection models; intra-peritoneal foreign body infection models; urinary tract infection models; ear, nose and throat infection models; respiratory tract infection models and osteomyelitis infection models have been used for the study of these infections.^74^ The use of mammalian model possess some difficulties that has made researchers to switch over to the non-mammalian model system such as *Drosophila melanogaster, Caenorhabditis elegans* or *Danio rerio*.^82^ The advantages of these models reside in the very short generation time and their lower cost. Moreover their small sizes provide the ease to maintain them in microtitre plates thus making it easier for high throughput screening of biofilm formation.

## Methods for quantification and structural assessment of biofilm

Biofilm production can be assessed by several methods. The standard assay for screening the presence of biofilms is crystal violet (CV) assay by quantifying the dye bound to cells on polystyrene and other hydrophobic substratum. However, the limitation of crystal violet assay includes its indirect nature, and requires repeated washings, which may cause loss of cells and above all the biofilm has to be disrupted.^83^ Tissue Culture Plate (TCP) method^84^ is also one of the most commonly practiced standard method and a more reliable process as compared with Congo Red Agar method (CRA)^85^ and Tube method (TM).^84^ Other methods for detection of biofilm include bioluminescent assay,^86^ piezoelectric sensors,^87^ and Percentage Transmission (%T) method.^88^ The advances in biofilm imaging technology have been proved to be very crucial to understand the complexity and dynamics of biofilms. These optical techniques include fluorescent microscopic examination,^89^ scanning electron microscopy (SEM),^80^ confocal scanning laser microscopy (CSLM), light microscopy, infrared spectroscopy, reflectance spectroscopy and optical fluorometry, which can be used to check the existence and visualize the 3D structure of biofilm.^90-92^ However, SEM is expensive and quantitation of the biofilm is difficult. Information on biofilm heterogeneity and cell localization can be obtained via fluorescence staining coupled with CSLM followed by high-speed computing. Reflectance assay is a semi-quantitative, inexpensive, and nondestructive optical assay for biofilms on abiotic surfaces and to some extent on biotic surfaces. This assay is capable of examining the status and morphology of biofilm formation and also reveals the biofilm forming ability of bacteria.^93-95^ To characterize the chemistry of biofilm NMR and FTIR can be taken into use. Nuclear Magnetic Resonance (NMR) imaging results demonstrate the water dynamics, molecular dynamics and biomolecule diffusion within biofilms^96,97^ and analysis of Fourier Transform Infrared Spectroscopy (FTIR) along with Raman imaging of biofilm permits the characterization of extracellular and cellular components. Raman imaging generates detailed chemical image based on sample's Raman spectra. Raman spectrometry along with CSLM provides information on the spatial distribution of biomass, water and chemical composition of *P. aeruginosa* biofilms.^98,99^ One of the most important quantitative assay is XTT ((2,3-bis (2-methoxy-4-nitro-5-sulfophenyl)-5-[(phenylamino) carbonyl]-2H- tetrazolium hydroxide) reduction assay, where the tertazolium dye, XTT is converted to water soluble colored formazan due to normal metabolic activities of cell.^88,100^ It not only facilitates the study of intact biofilm but also investigates biofilm drug susceptibility keeping the biofilm structure undisrupted.^101^

For the routine diagnosis of biofilm infections several microbiological techniques are commonly used. Proper sonication of indwelling devices is found to be an efficient technique for the detection of biofilm followed by16S rRNA sequence detection for identifying the strain of the organism involved.^102,103^ In some cases, the identification of biofilms can be done by examining blood leukocyte count, C-reactive protein, interleukin-6 and pro-calcitonin level.^104,105^ In addition, fluorescent *in situ* hybridization (FISH) is used for the diagnosis of biofilm infections in cystic fibrosis and chronic wounds using either traditionally labeled DNA probes or the Peptide Nucleic Acid (PNA) probes, the latter having better attributes thereafter providing information on community structure of biofilms.^106-112^ To measure anti-biofilm activity, viability and matrix biomass is assessed. For this, resazurin and crystal violet staining are performed sequentially in the same plate. Wheatgerm agglutinin-Alexa Fluor 488 fluorescent conjugate are generally used to stain the matrix. It is essential to measure the biofilm matrix, biomass and viability to investigate the efficiency of antibiotic treatment.^113^ The different methods of biofilm detection with their principles are enlisted in the Table 1.

**Table 1.** Different methods used for biofilm quantification.

S. No.	Methods of biofilm detection	Principle	References
1	Tissue culture plate method	TCP method is a standard method for biofilm detection. It simply involves the staining of cells with crystal violet dye.	^84^
2	Tube method	Crystal violet staining → A visible lining appears on the bottom and wall of tube → confirms biofilm formation	^84^
3	Congo red agar method	Congo red staining → black colonies in crystalline form appears → confirms biofilm production	^85^
4	Bioluminescent assay	This assay is based on the signaling based detection of metabolically active cells. It involves the catalysis of ATP and luciferin by luciferase.	^86^
5	Crystal violet assay (CV assay)	The CV assay quantifies the dye bound to biofilm. It actually quantifies all biomass (live, dead and also matrix of biofilm)	^83^
6	XTT reduction assay	It is mainly used for the quantification of Candida biofilms. The reagent XTT: (2,3-bis (2-methoxy-4-nitro-5-sulfophenyl)-5-[(phenylamino) carbonyl]-2H-tetrazolium hydroxide is involved here. XTT is an age dependent assay hence mature biofilms gives low intensity of color with XTT due to less viability of cells.	^88,100^
7	Scanning Electron Microscopy	This is used to study the morphology of bacteria attached on the surface and for enumeration of adhered bacteria.	^253^
8	Fluorescent *In-situ* Hybridization (FISH)	This is used to visualize the patterns of microbial colonization and the composition of microbial communities.	^108^
9	Confocal scanning laser microscopy	This gives the 3-dimensional view of the microbial community. It can show the focused part as well as the part out of focus.	^90-92^
10	Infrared spectroscopy	This technique is used to study molecules such as proteins, polysaccharides, metabolites essential for biofilms. It also gives the information about different hydrogen bonding states of water. Using Attenuated Total Reflectance Infrared (ATR-IR spectroscopy, the early biofilm development stages including bacterial attachment and growth can be studied.	^94,95^
11	Piezoelectric sensors	These monitors the shift in the frequency due to accumulation of mass on the surface of sensor	^87^

**Table 2.** Different anti-biofilm molecules and their target bacteria.

S.N.	Anti-biofilm molecules	Source	Target bacteria	MIC/MBC/MBIC/IC_50_ values	Reference
1.	Epigallocatechin gallate (EGCG)	*Camellia sinesis* (Green tea)	*Acinetobacter baumannii*,	MIC = 64–512 µg/ml	^254^
			*Pseudomonas aeruginosa*,		
			*Staphylococcus aureus*,	MBC = 64–1024 µg/ml	
			*Escherichia coli*,		
			*Stenotrophomonas maltophilia*		
2.	Ellagic acid	*Camellia sinensis*	*Streptococcus dysgalactiae*	MIC=4µg/ml	^245^
3.	Esculetin	*Santolina oblongifolia, Alchemilla speciose, Tagetes lucida*	*S. aureus*	MIC >512 µg/ml	^245^
4.	Fisetin	*Fragaria ananassa, Malus domestica, Vitis vinifera, Allium cepa, Solanum lycopersicum, Cucumis sativus*	*S. aureus*	MIC =64 µg/ml	^245^
			*Streptococcus dysgalactiae*	MIC = 64 µg/ml	
5.	Reserpine	*Rauwolfia vomitoria, Rauwolfia serpentine*	*Klebsiella pneumoniae*	MIC= 1000 µg/ml MBIC = 15.6 µg/mL	^255^
6.	Quercetin	*Usnea longissima*	*P. aeruginosa*,	MIC= 80 µg/ml	^256^
			*K. pneumoniae*		
7.	Linoleic acid	*Hydrastis canadensis*,	*K. pneumoniae*	MIC = 250 µg/ml MBIC = 31.2 µg/ml	^255^
		*Coptis chinensis, Berberis aquifolium, B. vulgaris, B. aristata*			
8.	Berberine	*Berberis aquifolium, B. vulgaris, B. aristata*	*K. pneumoniae*	MIC = 2000 µg/ml MBIC = 62.5 µg/ml	^255^
9.	Chitosan	Chitin	*K. pneumoniae*	MIC = 500 µg/ml MBIC= 62.5 µg/ml	^255^
10.	Eugenol	*Ocimum* plants,	*K. pneumoniae*	MIC = 250 µg/ml MBIC = 62.5 µg/ml	^255,257^
		*Syzigium aromaticum*			
			*S. mutans*	MIC = 0.3125 µg/ml	
11.	Curcumin	*Curcuma longa*	*K. pneumoniae*	MIC = 12500 µg/ml MBC = 250 µg/ml	^255^
			*Helicobacter pylori*	MBC = 8 µg/ml	
12.	Synthetic halogenated furanone (F-56)	Derived from natural furanone	*P. aeruginosa*,	—	^123^
			*Serratia liquifaciens*		
13.	Peptide 1018	*-*	*P. aeruginosa*	MIC = 64 µg/ml	^134^
				MBIC_50_ = 5 µg/ml	
				MBIC_100_ = 10 µg/ml	
			*E. coli*	MIC = 32 µg/ml	
				MBIC_50_ = 8 µg/ml	
				MBIC_100_ = 10 µg/ml	
			*A. baumannii*	MIC = 128 µg/ml	
				MBIC_50_ = 2 µg/ml	
				MBIC_100_ = 10 µg/ml	
			*K. pneumoniae*,	—	
			*S. aureus*,		
			*S. typhimurium*,		
			*Burkholderia cenocepacia*	MIC>256 µg/ml	
				MBIC_50_ = 2 µg/ml	
				MBIC_100_ = 10 µg/ml	
14.	CFT073 group-II capsular polysaccharide (Serotype K2)	Produced by extra intestinal *E.coli* of phylogenetic group B2 or D	*E. coli*,	—	^225^
			*P. aeruginosa*,		
			*K. pneumoniae*,		
			*S. aureus*		
15.	Pel polysaccharide	*P. aeruginosa*	*S. aureus*	—	^225,229,258^
16.	Psl polysaccharide	*P. aeruginosa*	*S. aureus*	—	^225,229,258^
17.	Sophorolipid (Biosurfactant)	Produced on microbial cells or excreted extracellular hydrophobic and hydrophilic moeities	*Cupriavidus*	5%(v/v)	^191^
			*necator*,		
			*Bacillus subtilis, S. aureus*		
18.	Colistin (PolymixinE)	*Paenibacillus polymyxa*	*S. maltophilia*	MIC = 158 µg/ml	^254^
				MBC = 256 µg/ml	
	Polymyxin B	*—*	*P. aeruginosa*,	MIC = 158 µg/ml	^259^
			*S. aureus*,		
			*E. coli*	MBC = 256 µg/ml	
19.	**Lantibiotics**: Nisin	*Lactococcus lactis*	*S. aureus*,	—	^189,190^
			*Staphylococcus epidermis*		
	Subtilin	*B. subtilis* strain ATCC6633	*Lactococcus lactis*	MIC=1µg/ml	
	Epidermin	*Staphylococcus epidermidis Tu3298*	*Lactococcus lactis*	MIC = 4–8 µg/ml	
	Gallidermin	*Staphylococcus gallinarum Tu3928*	*S. aureus*	MIC = 0.5µg/ml	
			*S. epidermidis*	MIC = 2.0µg/ml	
20.	**Antimicrobial peptide (AMP)**: LL-37	Human cationic host defense peptide	*P. aeruginosa*,	MIC = 0.5 µg/ml	^259,260,171,179,201-203^
			*S. aureus*,		
			*E. coli*		
	Lytic peptide (PTP-7)	Synthetic analog from Gaegurin 5	*P. aeruginosa*,	MIC = 2–16 µM	
			*S. aureus*,		
			*E. coli*		
	Sushi peptides	Derived from sushi-3 domain of Factor C, which is a LPS-sensitive serine protease of horseshoe crab coagulation cascade	*P. aeruginosa*,	—	
			*S. aureus*,		

			*E. coli*		
	PMAP-23	Cathelicidin-derived peptide identified from porcine leukocytes	*P. aeruginosa*,	—	
			*S. aureus*,		
			*E. coli*		
	PR-39	Isolated from the pig's small intestine	*P. aeruginosa*,	MIC = 0.94 µM	
			*S. aureus*,		
			*E. coli*		
	Buforin-II	Derived from Buforin-I (stomach tissue of *Bufobufo gargarizans*)	*P. aeruginosa*,	MIC = 0.25–4.0 µg/ml	
			*S. aureus*,		
			*E. coli*		
	Indolicidin	From cytoplasmic granules of bovine neutrophils	*P. aeruginosa*,	MIC = 50 µg/ml	
			*S. aureus*,		
			*E. coli*		
	Pyrrhocoricin		*P. aeruginosa*,	IC_50_<0.3 µM	
			*S. aureus*,		
			*E. coli*		
	Microcin B17	Post-translationally modified peptide that is produced by *E. coli* containing the plasmid-borne MccB17 operon	*E. coli*	IC_50_ = 0.9 µM	^261^
21.	**Chelating agents**: (a)Sodium citrate (b)Tetrasodium EDTA (c) Disodium-EDTA		*Staphylococcus* species,	MIC ≥ 0.5%	^260^
			*P. aeruginosa*		
22.	Tannic acid	*Caesalpinia spinosa, Rhus semialata, Quercus infectoria, Rhus coriaria*	*S. aureus*	—	^146^
23.	**Enzymes**: Deoxyribonuclease I, glycoside hydrolase (dispersin B)		*Staphylococcus and Enterococcus*	—	^121,260^
24.	Bacteriophage-encoded endolysin (PlyC)		*Streptococcus pyogenes*	MIC = 0.04–0.08 µg/ml	^150^
				MBC = 0.02–0.08 µg/ml	
				MBIC = 1.25–5 µg/ml	
25.	Silver		*P. aeruginosa, S. proteamaculans*	MBIC = 100000– 150000 µg/ml	^193^
26	Octenidine hydrochloride		*P. aeruginosa*,	—	^193^
			*S. aureus*		
27	Chlorhexidine		*P. aeruginosa*,	—	^193^
			*S. aureus*		
28	Cadexomer iodine		*S. aureus*	—	^193^
			*P. aeruginosa*		
29	Polyhexamethylene biguanide		*P. aeruginosa*	—	^193^
30	Usnic acid	A secondary lichen metabolite	*S. aureus, C. albicans*	—	^132,175^

**Table 3.** Mechanisms followed by different Anti-biofilm molecules.

S. N.	Mechanism of action	Molecules associated	Reference
1.	Inhibition of AHL-mediated quorum sensing pathway	Halogenated furanone compounds, Quercetin	^123,256^
2.	Inhibition of (p)ppGpp regulated stringent response	Peptide-1018, Peptide-1038	^134,140^
3.	Dispersion of Extracellular Polymeric Substance (EPS) of biofilm	Deoxyribonuclease I and glycoside hydrolase dispersin B	^259^
4.	Cleavage of peptidoglycan	Tannic acid, Endolysins (PlyC), Epigallocatechin gallate (EGCG)	^146,150,159^
5.	Biofilm disassembly	A cyclic autoinducing peptide (AIP), Nuclease, extracellular proteases (eg. sarA, sigB, *Esp*), antiamyloid molecules (AA-861, parthenolides), D- Tyrosine, Ethyl-pyruvate	^77,160,168,172^
6.	Neutralization/disaggregation of LPS	Polymyxin (B and E), Gramicidin S, Sushi peptides, PMAP-23	^177,180,262^
7.	Alteration of membrane permeabilization	Lantibiotics (nisin, gallidermin), Lytic peptides (PTP-7), Sophorolipids, Polyhexamethylene biguanide, Chlorhexidine, Pentasilver hexaoxoiodate	^179,186,191,192^
8.	Inhibition of cell division or cell survival	Pyrrhocoricin, Microcin B17	^194,198^
9.	Inhibition of macromolecule synthesis and adhesion of cells	Buforin II, PR-39, Indolicidin, LL-37, Bacteriocins, Cadexomer iodine, Mannosides, Pilicides	^39,193,201,202,204,209,221,222^
10.	Inhibition of biofilm by polysaccharides	EPS273, Psl and Pel, K2, PAM galactan, A101, PslG, Polysaccharides of algae, plants and animals	^224,225,227,228,230,235^
11.	Inhibition of c-di-GMP signaling system	LP 3134, LP 3145, LP 4010, LP 1062, ebselen, ebselen oxide Desformylflustra bromine	^6,238,239^
12.	Inhibition of curli biosynthesis	Analogs of FN075 and BibC6 of ring-fused 2-pyridones	^169^

## Strategies to combat biofilm formation

Since biofilm formation contributes to the bacterial pathogenicity and resistance toward antibiotics, there must be certain strategies to deal with this problem. Recently, Wu et al has reported the use of foreign bodies as a major cause of increase in biofilm infections.^6^ So, to treat such biofilm associated infections it is imperative to remove the indwelling medical equipment followed by replacement with new uninfected ones along with sensitive and aggressive administration of antibiotics. Moreover, the implant removal should be timed properly so that the new or replaced implant does not get infected when inserted in the patient's body. In cases where removal is not possible a long-term administration of antibiotics is recommended so as to avert the biofilm from growing. According to the previous reports, the premature biofilms can be treated more effectively with antibiotics than that of the mature biofilms. However, the inefficiency to diagnose premature biofilms in the body is the most crucial reason for the occurrence of clinical conditions, which are mostly related to mature form of biofilms.^9-11,114^ The antibiotic used for the treatment of biofilms should be legitimately selected on the basis of sensitivity as well as the capacity to penetrate properly through the biofilm matrix.^6^ It is evident from the previous studies that bacteria underneath biofilms are more resistant to antibiotics than their planktonic counterparts. Therefore, the use of combinatorial therapy is more preferable instead of antibiotic monotherapy.^115^ The combination of agents is advantageous due to different functioning of the individual agents; for example, one may be effective against dormant cells and other against the growing cells. Moreover, the therapy also requires proper dispensation of antibiotic in terms of dosages and duration. The discovery of antifouling or antimicrobial surfaces can be another possible approach to prevent biofilm formation.^116^ Polymeric hydrophilic coatings such as PEG are used for building antifouling surfaces as they minimize or hamper the microbial adhesion. Building of antimicrobial surfaces involves impregnation with antibiotics or disinfectants, mainly polyurethane polymers, which are loaded with different antibiotics.^117,118^ Coating with nanoparticles such as silver nanoparticles, antioxidant nanoparticles can also be used for the prevention of biofilm formation.^69,119^ However, the coating strategy has encountered difficulties as the surface quickly gets eroded and hence becomes available for the formation of biofilms. Photodynamic therapy (PDT) has potential applications in prevention of wound biofilm infections. Here, a photoactive dye is used followed by irradiation in the presence of oxygen, thus killing the bacteria.^120^ During therapy, proper care should be taken that patient's eyes are not exposed to laser light. With respect to photosensitizer and photochemical reactions, it is very crucial that the therapy should be used carefully to stain and kill the bacterial cells only without affecting the surrounding tissues of the patient's body. Another uprising strategy is the use of effective anti-biofilm molecules or the biofilm dissolving substances.^121^ The anti-biofilm molecules interfere with bacterial signaling pathways in both Gram-positive and Gram-negative bacteria. The anti-biofilm molecules may be any enzyme, a peptide, an antibiotic, polyphenols etc. In the present review, we have discussed about the different anti-biofilm molecules discovered against different bacterial infections. We have also highlighted the mechanism of action of different available anti-biofilm molecules. This review will help to understand the targets for anti-biofilm molecules and help researchers working in the discovery of new antibiotics for Gram-negative bacteria.

## Anti-biofilm molecules and their mechanism of action

Anti-biofilm molecules belong to diverse compounds thereby inhibiting the biofilm formation. The identified anti-biofilm compounds are mainly isolated from the natural sources,^122^ some synthetic compounds, chelating agents, and lantibiotics also have been found to possess anti-biofilm activity. The different anti-biofilm molecules along with their target microorganisms are listed in the Table 2. These anti-biofilm molecules follow different mechanisms to inhibit biofilm formation in different bacteria as listed in Table 3.

## Inhibition of AHL-Mediated quorum sensing

N-acyl homo-serine lactones (AHLs) are used as the signaling molecules by numerous bacteria especially, Gram-negative bacteria during quorum sensing to control their population density as well as facilitate swarming motility. These signaling molecules vary in their length, substitutions on acyl side chains^123^ and are synthesized by a LuxI-type synthase. Binding of these molecules at certain critical concentrations, to a cognate LuxR-type transcriptional activator protein regulates the target gene expression.^124,125^ A secondary metabolite derivative, synthetic halogenated furanone (Fig. 2) compound is derived from natural furanone produced by the Australian macroalga, *Dilsea pulchra*. This compound has the capability to interfere with bacterial signaling processes and motility of swarm cells. It was also hypothesized that similarity in the structure of *D. pulchra* furanones and AHL molecules is responsible for affecting the interaction of putative regulatory protein with AHL molecules via binding competitively to the receptor. Furanones inhibit surface aggregation traits in ecologically relevant bacteria in ecologically relevant concentrations.^126^ Transcription of *lasB-gfp* (ASV) reporter fusion, regulated by quorum sensing is interfered by furanone 56 decreasing the extracellular chitinase and elastase activity and having almost no effect on the growth of the bacteria or in the protein synthesis. Studies suggested that furanone targets the *rhl* system which is involved in the quorum sensing and also penetrates the biofilm matrix of *P. aeruginosa* thereby affecting the expression of genes related to quorum sensing bioflm maturity. This molecule alters the structure of the biofilm which facilitates bacterial detachment at an increased rate and results in the loss of biomass of the bacteria from substratum.^123^ It was also discovered that furanone mediates displacement of AHL molecules from Lux R,^127^ which suggested the competence of furanone with the cognate AHL signal for the LuxR receptor site. At present, there are several experimental evidences which support the observations about furanones such as, repression of AHL-dependent expression of bioluminescence,^127^ inhibition of production and pathogenesis of AHL-controlled virulence factor,^123,128^ and inhibition of quorum sensing–controlled luminescence.^129^ Some polyphenols (like EGCG, tannic acid, ellagic acid) (Fig. 2) are believed to follow the similar mechanism to inhibit biofilm formation but due to their less efficiency than furanones, they are required in higher concentration.^38^ Quercetin (Fig. 2), a flavonoid, also influences quorum sensing, hence acts as an anti-biofilm compound against *S. aureus*. It inhibits alginate production in a concentration dependent manner; resulting into declination in the adherence during biofilm formation. It also reduces exopolysaccharide (EPS) production required for the initial attachment of bacteria and leads to induction of swarming motility.^130^ Apart from quercetin, 2 more synthetic flavanoids are also identified, which act as potential antimicrobial agent against the dispersed cells as well as biofilm of *S. aureus*.^131^ Some other reports also suggested that usinic acid show inhibitory effect on the *S. aureus* biofilm and affected the morphology of biofilm produced by *P. aeruginosa*. Researchers have hypothesized that this may be due to any interference in quorum sensing, but the exact mechanism of action is still unclear.^132^ Curcumin (Fig. 2), a phytochemical from the rhizome of Curcuma longa exhibit potent antibiofilm effect by the modulation of expression of genes involved in quorum sensing and related virulence factors like alginate production, and swarming motility.^133^

**Figure 1.**
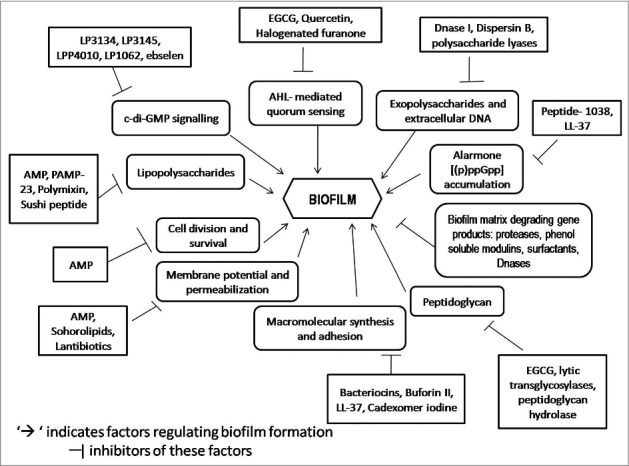
Schematic representation of overview of the targets of anti-biofilm molecules.

**Figure 2.**
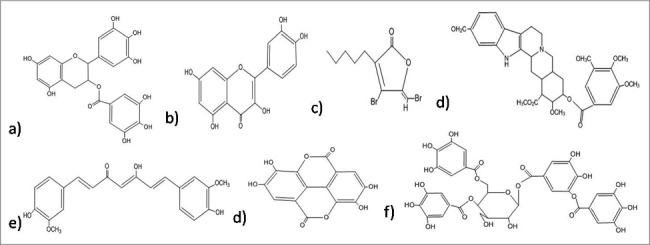
Structures of the anti-biofilm molecules that inhibit AHL-mediated quorum sensing. (a) EGCG^263^, (b) Quercetin^264^, (c) Synthetic halogenated furanone^265^, (d) Reserpine^266^, (e) Curcumin^267^, (f) Ellagic acid^268^, (g) Tannic acid^269^.

## Inhibition of stringent response by bacteria

**Figure 3.**
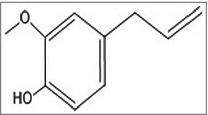
Structures of the anti-biofilm molecules that inhibit the stringent response. (a) Eugenol^270^.

A peptide named 1018-peptide works by inhibiting the alarmone accumulation, which is a part of stringent response by bacteria in response to nutritional stress. During their stress conditions bacteria synthesize alarmones, namely; guanosine tetraphosphate and guanosine pentaphosphate, collectively termed as (p)ppGpp.^134,135^ Generally, 2 enzymes regulate the (p)ppGpp metabolism: the (p)ppGpp-synthetase/hydrolase, RelA (orRsh) with dual function and the small alarmone synthetase, RelQ. Previous works by researchers suggest that during stress conditions, rapid accumulation of (p)ppGpp is regulated by RelA whereas RelQ is found responsible for low level of expression of (p)ppGpp under no stress conditions. The absence of (p)ppGpp, in the case of a ΔrelA-ΔrelQ double mutant strain, leads to reduced antibiotic tolerance and attenuated virulence.^136^ Studies also revealed that any change in (p)ppGpp pool can severely affect the bacterial biofilm formation, development and maintenance of its stable form *in-vitro*. The RelA-mediated stringent response and helps in the cell survival as during starvation conditions it can optimize gene expression for growth and survival. Some results prove the importance of the (p) ppGpp synthetase, RelQ in biofilm homeostasis. In case of ΔrelA ΔrelQ strain, there occurs some severe defects during the formation of biofilm, its maturation and viability due to uncontrolled consumption of energy resource, NAD/NADH ratio imbalance, or amassing of the metabolic end products.^137^ Hence, the amphipathic cationic peptide 1018 must establish direct contact with (p)ppGpp by crossing through the cell membrane and reaching protoplasm. The peptide disrupts the biofilm in 3 possible manners. First, it prevents formation of biofilm when added before initiation step. Second, at much specified low concentration it disrupts and kills the bacteria present in biofilm without affecting the planktonic cells. Third, it can disperse mature biofilm which is 2 d old.^134^ It was also reported to exhibit significant synergistic effect with antibiotics against biofilm formed by different bacteria.^138^ The peptide 1018 and its derivatives HE4 and HE10 are found to be active against *P. aeruginosa* and *B. cenocepacia* at concentrations much below the MIC of their planktonic growth. In contrast to the parent molecule 1018, these derivatives exhibit equal or decreased level of anti-biofilm activity against biofilm formed by *P. aeruginosa*, but increased activity against methicillin resistant *S. aureus* biofilms.^139^ Peptide 1037 was also evident to reduce biofilms formed by many Gram- positive and Gram-negative bacteria.^140^ Another peptide named, Peptide 1038^140^ induce twitching motility (which destroy biofilm), and inhibit adhesion and quorum sensing of *Pseudomonas* involved in biofilm formation. A secondary metabolite from *Syzigium aromaticum*, eugenol (Fig. 3) treatment to *S. mutans* causes the downregulation of gene, *relA*, involved in the control of stringent response in biofilm formation as well as acid tolerance.^141^

## Dispersion of extracellular polysaccharide substance of biofilm by enzymes

The Extracellular Polysaccharide Substance (EPS) of the biofilm protects the microorganisms from various antimicrobial agents. The disorganization of the EPS would expose the released as well as residual biofilm cells to these agents. There are certain enzymes such as polysaccharide lyases and DNases that are capable of disrupting the exo-polysaccharides.^142^ Likewise, DNase I and Dispersin B are the major enzymes that functions as potential anti-biofilm agents.^143,144^ DNase I is capable of digesting the extracellular DNA (eDNA) which is present within the biofilm structure whereas Dispersin B, a glycoside hydrolase works by cleaving polymers of β 1–6 N-acetylglucosamine (PNAG), an extracellular polysaccharide substance which facilitates aggregation of bacteria. Also, it can disperse EPS layers present on medical devices.^143,144^ These biofilm-dispersing enzymes are more efficient when administered in combination with antimicrobial agents in killing the bacteria embedded in the EPS.^145^

## Cleavage of peptidoglycan

The cleavage of peptidoglycan, which is present in the cell wall of most of the bacteria inhibit biofilm generation. Tannic acid, a polyphenolic compound, inhibits biofilm formation without affecting bacterial growth in *Staphylococcus aureus*.^146^ The mechanism of action was found to depend on the putative lytic transglycosylase, an immune-dominant *Staphylococcal* Antigen A (IsaA), which acts by cleaving peptidoglycan.^147^ These transglycolases are lysozyme-like enzyme which catalyzes the cleavage of the β-1,4 glycosidic bond between N-acetyl muramic acid (MurNAc) and N-acetyl glucosamine (GlcNAc).^148^ Tannic acid inhibits the formation of biofilms by increasing the extracellular level of IsaA.^146^ Cleavage of peptidoglycan reduces biofilm formation by several ways, such as; it alters the composition of proteins and teichoic acids present on the cell wall. Peptidoglycan cleavage may also result in the release of signaling molecules^149^ that can modulate the biofilm-related gene expression. Bacteriophages encode a unique class of peptidoglycan hydrolases referred as endolysins,^150^ which digest the cell wall of bacteria so as to release progeny bacteriophage. Endolysins usually work in a species-specific manner. They bind with the cell wall and cleave it, which ultimately leads to hypotonic lysis and bacterial death.^151^ Endolysins work on the multiple antibiotic resistant strains. *PlyC*, a specific streptococcal bacteriophage endolysin,^152-156^ functions by disrupting the *in-vitro* biofilms. This bacteriophage therapy requires the knowledge of the bacteria causing infections, for which specific bacteriophages are needed to be design properly. Another molecule, epigallocatechin gallate, a polyphenol, inhibits bacteria by causing the cell wall damage via binding with the peptidoglycan,^157,158^ thus interferes with initial docking phase (mainly due to hydrophobic interactions) of biofilm formation.^159^

## Inhibition through biofilm disassembly

Biofilm disassembly is a multistep process that involves deterioration of the extracellular matrix and change in the physiology of cell which prepares them to sustain in conditions persisting beyond the boundary of the biofilm.^160^ Several bacterial species takes into account a primary mechanism of disassembly of biofilms that include the production of extracellular enzymes or surfactants that leads to degradation as well as solubilization of adhesive components in the matrix of biofilm. The matrix keep the cells enclosed within the biofilm colony, thus, upon its deterioration cells get detached from this colony and are released in the environment. The active biofilm dispersal is mediated by certain matrix-degrading gene products such as proteases, deoxyribonucleases (DNases) and surfactants.

An accessory gene regulatory (agr) system present in several bacteria, control the synthesis of biofilm matrix degrading enzymes. The agr system is mediated by a cyclic auto-inducing peptide (AIP). At critical threshold concentration (in the low nanomolar range), a 2-component signal transduction cascade is activated by AIP which results into the generation of virulence factors.^161^ The extracellular proteome of agr includes several proteases and small pore-forming toxins known as phenol-soluble modulins (PSMs).^29,162,163^ Thus, activation of the agr system prevents biofilm maturation, performing an inhibitory role.^29^

The production of extracellular proteases (eg. sarA, sigB, Esp) has been found to be associated with the biofilm disassembly mechanism.^162,164,165^ During biofilm disassembly, nuclease acts as an endogenous mediator. It is an effective DNase (a thermonuclease or micrococcal nuclease) that helps in the separation of cells from biofilms.^166^ In some species, when the DNases and restriction enzymes are added exogenously, the biofilms get readily dispersed from microplate wells, which indicates that extracellular DNA (eDNA) is a major biofilm matrix moieties there.^144,166^ The amyloid-like fibers and the secretion of TasA protein have also been reported to play an important role in the formation of biofilms.^167,168^ The detachment of these amyloid-like fibers from the cell surfaces lead to the disassembly of biofilms.^168^ To screen the anti-amyloid activities of molecules, *B. subtilis* biofilms, being the simplest biologic system are mainly preferred. AA-861 and parthenolide exhibit inhibitory properties against biofilms by *B. subtilis, E. coli* and *Bacillus cereus* by interfering with the polymerization of TasA into amyloid-like fibers.^168^ *E. coli* and other species of *Enterobacteriaceae* produce functional amyloid fibers named curli. Type 1 pili and curli plays significant roles in promoting biofilm in *E. coli*. Some earlier studies suggested that 2 analogs of FN075 and BibC6 of ring-fused 2-pyridones are peptidomimetic that target the protein–protein interactions in macromolecular assembly, blocking the synthesis of curli in *E. coli*. Bacterial virulence is significantly attenuated in a mouse urinary tract infection model when *E. coli* was pretreated with FN075.^169^ Additionally, Connolly et al.^170^ and Park et al.^171^ reported the use of cysteine protease SpeB and proteases from Group A *Streptococcus* and *P. aeruginosa*, respectively, for the biofilm dispersal.

D-tyrosine leads to a significant decrease in cell attachment thus, preventing the formation of biofilm. It also causes biofilm disassembly at a very low concentration in both *B. subtilis* and *P. aeruginosa*. Impact of D-tyrosine on EPS production and extracellular protein is concentration specific and varies greatly in Gram-positive and Gram-negative bacteria. The concentration of extracellular proteins increased in *B. subtilis* biofilms and reduced in the biofilms of *P. aeruginosa*. Moreover, EPS production increased when *P. aeruginosa* was treated with low concentration of D-tyrosine and decreased at higher concentrations but no change was observed in *B. subtilis*.^172^ Hence, it is very crucial to decide the dosage carefully before recommending D-tyrosine for treatment of biofilms. *In-vitro* studies also suggested that D-histidine, D-cysteine and D-tryptophan inhibit 35–86% biofilm formation in *A. baumannii* at a very low concentration of 2 mM and D-cystine, D-tryptophan and D-tyrosine inhibit 10–30% biofilm formation in *P. aeruginosa* at 4 mM. However, it is also demonstrated in this study that significant effects of D-amino acids were not observed *in-vivo* as the effective *in-vitro* concentration produced toxic effects and even fatal when tested on mouse models. The use of D-amino acids with antibiotics should be further investigated.^173^ Some recent *in-vitro* and *ex-vivo* studies suggested nagZ, a protein involved in peptidoglycan recycling, also reduces preformed biofilm in *Neisseria gonorrhoeae* but the exact mechanism of biofilm dispersal is still unclear.^174^

**Figure 4.**
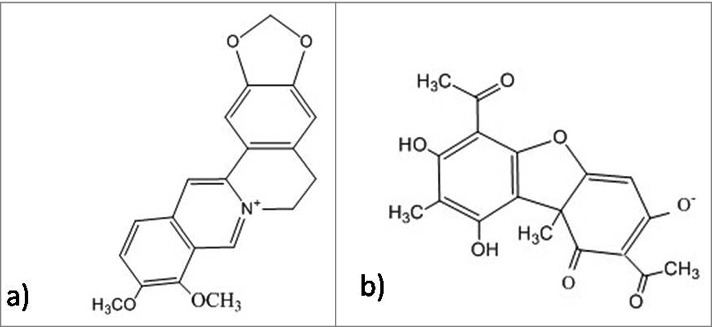
Structure of anti biofilm molecules that disassemble the biofilm. (a) Berberine^271^, (b) Usnic acid^272^.

A lichen secondary metabolite, usnic acid (Fig. 4), has the potential to inhibit 65% biofilm formation and yeast to hyphal transition. This compound not only prevents adhesion but also reduces various sugars in EPS. Light microscopic studies revealed that usnic acid stops the transition from yeast to hyphal state thereby reducing the thickness of matured biofilm.^175^ Some other reports also suggested that usnic acid show inhibitory effect on the *S. aureus* biofilm and affected the morphology of biofilm produced by *P. aeruginosa*. Researchers have hypothesized that this may be due to any interference in quorum sensing, but the exact mechanism of action is still unclear.^132^

In some recent studies, ethyl pyruvate (EP), an anti-tumor and anti-trypanosome drug, has been reported to possess significant antimicrobial and anti-biofilm activity. The architecture of the biofilm matrix is especially stabilized by DNA and negatively charged polymeric sugars, bridged by Ca^2+^ ions. EP acts as a potential Ca^2+^ ions chelating agent due to its dicarbonyl structure, which destabilizes the biofilm matrix. Moreover, this specific structural element plays a crucial role in inhibiting enzymes pyruvate kinase of glycolytic and glyoxalase-1 of para-glycolytic pathway. This explains the antimicrobial activity of EP. The study reveals EP to be better anti-biofilm agent for being tissue protective, showing no side-effects in clinical studies, harmless to symbionts, inhibiting a broad spectrum target such as bacteria, fungi, parasites and mold, lower chance of developing resistance, inhibiting adhesion and maturation of biofilm and the dissolution of pre-formed biofilm matrix.^77^

## Neutralization or disassembly of lipopolysaccharides

**Figure 5.**
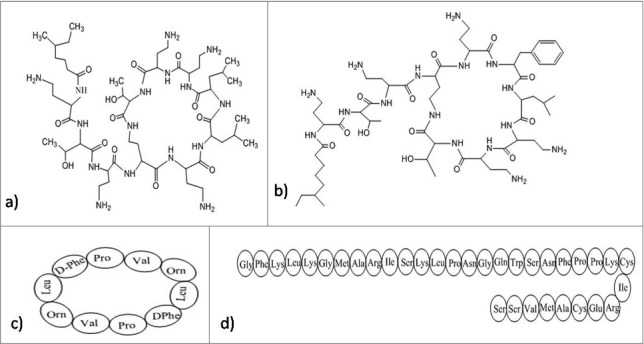
Structures of the anti-biofilm molecules that inhibit lipopolysachharides. (a) Colistin (Polymixin E)^273^, (b) Polymixin B^274,275^, (c) Gramicidin S^276^, (d) Sushi peptide (S1 domain)^181^.

The antimicrobial peptide (AMP) is an alternative of conventional antibiotics and is considered as an effective anti-biofilm agent. AMPs are evolutionary conserved proteins with low molecular weight and exhibits antimicrobial activity against fungi, bacteria and viruses. They are generally positively charged and contain both hydrophilic and hydrophobic sides which make them capable of penetrating the lipid bilayer as well as solubilizing in aquatic environment.^176^ Antimicrobial peptides usually bind electrostatically with lipopolysaccharides (LPS) involving interaction between 2 cationic amino acids (lysine and arginine) and their respective head groups. The complex is stabilized through hydrophobic interactions between the hydrophobic amino acids of the peptide and fatty acyl chains of LPS^177,178^ and resulting into destabilization of lipid head groups by multiple pore formation, thereby disrupting the integrity of cellular membrane. PTP-7, an example of lytic peptide is a synthetic analog from Gaegurin 5. Despite being a cationic peptide, its activity is not affected by acidic pH, negatively charged extracellular polysaccharides in biofilm matrix or high metal ion concentrations. Rather it is capable of entering deep in the biofilm and kill bacteria very efficiently.^179^ Polymyxins, especially polymixin E or colistin and polymixin B (Fig. 5) (pentabasic decapeptide antibiotic) bind to lipid A of LPS in Gram-negative bacteria making the outer membrane permeabilized. Along with this, Gramicidin S (Fig. 5) also disturbs the membrane integrity of the Gram-positive and Gram-negative bacteria. Both these cationic cyclic peptides possess specific targets in cell membrane leading to interference in the hydrophobic interactions at ligand binding sites of the enzymes. Further improvement of toxicity, structural analysis and clinical tests should be performed for using it clinically.^180^ Likewise, sushi peptides, a derivative of Factor C (Fig. 5) (LPS-sensitive serine protease of the horseshoe crab coagulation cascade) follow detergent-like mechanism for the disruption of LPS aggregates. They have LPS-neutralizing activity too. They act very specifically with palmitoyl-oleoyl-phosphatidylglycerol (POPG). Unsaturated POPG renders fluidity and ultimately increases the entry of peptides in lipid bilayer, completely disrupting membrane stability.^177,181^

## Alteration of membrane potential or membrane permeabilization

The alteration of membrane potential or membrane permeabilization is yet another mode of action of antimicrobial peptides. This results into disruption of the cytoplasmic membrane via pore formation either through a barrel-stave,^182^ a toroidal pore,^183,184^ or through a non-pore carpet-like mechanism^185^ that result into the efflux of intracellular materials.

**Figure 6.**
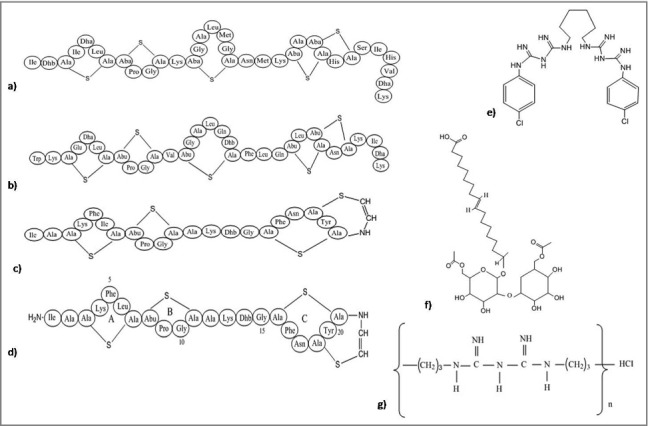
Structures of the anti-biofilm molecules that alter the membrane potential or membrane permeabilization. (a) Nisin^277,278^, (b) Subtilin^279^, (c) Epidermin^280^, (d) Gallidermin^281^, (e) Chlorhexidine^282^, (f) Sophorolipid^283^, (g) Polyhexamethylene biguanide^284^.

The lantibiotics are the class of the peptide antibiotics with ring structure, linked via thioester containing lanthionine and methylanthionine, or unsaturated amino acids dehydro alanine or 2-amino isobutyric acids. They are synthesized by ribosomes and modified post-translationally in Gram-negative bacteria and serve as anti-biofilm agents. These peptides comprises of an intra-molecular ring structure and can inhibit a wide-range of bacteria.^186^ These compounds pose an inhibitory effect on bacteria by damaging its bacterial membrane thus, inhibiting the production of enzymes. The most well known lantibiotic, nisin (Fig. 6), forms complex with lipid I and II thus, resulting in the inhibition of the cell wall biosynthesis.^187,188^ Nisin can also induce permeability to the cytoplasmic membrane by producing pores with short life-span.^186^ Subtilin (Fig. 6), another pore-forming lantibiotic structurally similar to nisin, acts by dissipating the transmembrane proton motive force resulting in the release of cytoplasmic solutes from *Staphylococcus simulans* and *B. subtilis* cells and from membrane vesicles. Subtilin binds to bactoprenyl pyrophosphate and causes membrane permeabilization in a lipid II-dependent fashion. *In-vitro* modifications were successfully used to insert thioester rings in various biologically active peptides. Clinical use of these modified lantibiotics can be ensured after proper *in-vivo* tests.^189^ Epidermin and gallidermin have the same putative lipid II binding motif as nisin; however, differing only in size with 22 amino acids, as compared with 34 in nisin. These 2 lantibiotics interfere with lipid II biosynthesis and interact with lipid-I, lipid-II and their intermediates that ultimately prove to be fatal for the bacteria. Studies show that gallidermin efficiently inhibits biofilm formation by *Staphylococci*, which might be due to repression of genes involved in biofilm formation, such as atl (major autolysin) and ica (intercellular adhesin). However, the effect of gallidermin on mature (24-h and 5-day-old) biofilm was significantly reduced.^190^

Biosurfactants are amphipathic molecules that possess antibacterial activity, inhibit bacterial cell-surface adhesion and hence disrupt biofilm. Sophorolipids are a class of biosurfactants that act against biofilms by increasing the permeability of membrane. In *B. subtilis*, sophorolipids (Fig. 6) disrupt the bacterial cells followed by release of an intracellular enzyme, malate dehydrogenase causing efflux of their cytoplasmic contents. They also inhibit biofilm produced by single as well as mixed culture of *B. subtilis* and *S. aureus* at very low concentration. This result indicated the use of sophorolipids as an adjuvant with other antibacterial agents that inhibit bacterial growth or disassemble the biofilm of some pathogens.^191^ Biofilm can also be destroyed by the use of polyhexamethylene biguanide (Fig. 6); a cationic antimicrobial agent disrupts the membrane and hampers the cell permeability without cell wall lysis. Chlorhexidine (Fig. 6) changes the osmolarity of the cell by binding with the negatively charged component. Compared to these 2, a silver compound penta-silver hexaoxoiodate (Ag(5)IO(6)) is much more efficient in killing of a broad spectrum planktonic organisms, inhibition of microbial adhesion to surface for longer time period and disassemble and eradicate mature biofilms of *C. albicans, P. aeruginosa*, and *S. aureus*. The reason behind the high efficiency may be due to structure of the nanomaterial, which possess silver in both cation and anion along with the protection of anion by iodate. This compound may consider to be used as a potent antimicrobial agent for disinfecting medical devices like catheters, implants, ventilators and wound dressing.^192^

## Inhibition of cell division and survival

**Figure 7.**
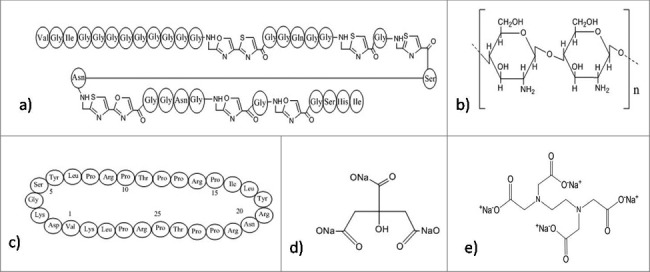
Structures of the anti-biofilm molecules that inhibit cell division and survival. (a) Microcin B17^285,286^, (b) Chitosan^200^, (c) Pyrrhocoricin^287^, (d) Sodium Citrate^288^, (e) Tetrasodium EDTA^289^.

Cell division is very crucial for the survival of bacteria in biofilm and their further spread to a new area. Silver accumulates within the intracellular vacuoles resulting in the damage of plasma membrane followed by alteration in the electric potential, thereby preventing cell division.^122,193^ Some antimicrobial peptides function by inhibiting cytoplasmic proteins, which have role in cell division and survival. These peptides penetrate into the cytosol of bacteria either by flip-flop method or channel formation in the outer membrane protein. Some antibacterial peptides are rich in proline such as pyrrhocoricin (Fig. 7),^194^ apidaecin^195^ and drosocin.^196^ All these peptides are capable to bind with multi-helical lid region of DnaK (a heat shock protein of bacteria) and interfere in the initiation step of chromosomal DNA replication. Moreover, they also interfere in the interaction of DnaK with DnaJ that causes bacterial death. Pyrrhocoricin enters into bacterial cytosol via C-terminus and the N-terminus is responsible for inhibition of ATPase activity of DnaK protein.^194^ In addition to this, proline-rich AMPs actively enter the bacterial cell and interfering in translation initiation via binding to the tunnel of ribosome.^197^ Microcin B17 (Fig. 7), a ribosomally synthesized antimicrobial peptide from *Enterobacteriaceae* inhibits DNA gyrase followed by inhibition of DNA replication. Also, it is the first peptide that has the capability to inhibit a type II DNA topoisomerase.^198^ Apart from this, chelating agents like EDTA (Fig. 7) are able to potentiate the cell wall, thereafter destabilizing the biofilms via sequestering iron, zinc, magnesium, and calcium. This makes them suitable for the management of biofilms.^199^ Chitosan, a natural polymer, due to its cationic nature has the capability to disrupt negatively charged cell membranes as soon as microbes settle on the surface.^200^

## Inhibition of adhesion molecule synthesis and function

**Figure 8.**
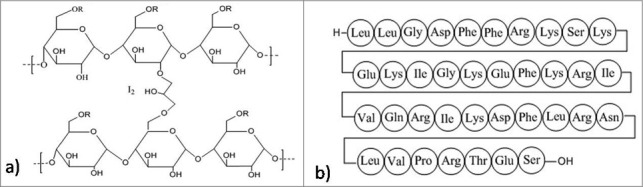
Structures of the anti-biofilm molecules that Inhibit adhesion molecule synthesis and function (a) Cadexomer iodine^290^, (b) LL-37^291^.

There are some classes of AMPs that exhibit killing of bacteria by direct interaction with nucleic acids without causing permeabilization of the membrane like, Buforin II.^201^ The antimicrobial peptide PR-39 (isolated from the pig's small intestine) can penetrate the outer membrane, and stop the synthesis of DNA and protein, the basic components of biofilms.^202^ Another peptide, indolicidin, permeabilizes the membrane without causing the lysis of bacterial cells. It also inhibits DNA synthesis,^203^ and exhibits binding specificity with DNA rather than RNA.^204^ Studies also reported that a cationic peptide LL-37 (Fig. 8) is present in human as host defense peptide, has the capacity to reduce the bacterial adhesion and promote stimulation of twitching motility mediated by type IV pili. It also stimulates the downregulation of genes related to quorum sensing.^39^ It was found active against *S. epidermidis* inhibiting the attachment of bacteria and thereby the formation of biofilms.^205^ Studies also revealed that citropin (from green tree frog Litoria citropa)^206^ and melimine (a non-hemolytic hybrid peptide)^207^ have potent s activities against *P. aeruginosa* and *S. aureus* and did not pose any toxic effect when tested in animal models. These molecules can be used to prevent bacterial adhesion on medical equipments like catheters and contact lenses. Another modified peptide, cadexomer iodine (Fig. 8), binds with the cytoplasmic membrane proteins followed by its penetration into the bacterial cell leading to the inhibition of protein synthesis and the disruption of lipid membrane as well as it interferes with the functioning of nucleic acids.^193^ Recent studies also demonstrate the ability of AMP to coat the bacteria or the surface of the biomaterials leading to the reduction of adhesion by bacteria as well as reduced biofilm formation.^208^ Bacteriocins like bovicin HC5 (produced by *Streptococcus bovis* HC5) and nisin are found to alter the hydrophobicity of the surfaces of S. aureus attachment, thus minimizing the adhesion to surfaces of food items, which is thought to be a better option than eradication of the already established biofilms. This facilitates long time storage and preservation of packaged food items.^209^ The pili or fimbriae are the long filamentous surface structures that enable bacteria to adhere to the host tissues and are also found to be involved in biofilm formation. PilB and PilA components of pili are important for biofilm formation but not PilC.^210^ Pili are classified into 2 groups. Type I pili comprises of mainly 2 components- FimA (major part) and FimH (minor part). FimH is a mannose-binding adhesion component which facilitates bacterial invasion.^211^ Most of the uropathogenic *Escherichia coli* (UPEC) possess type I pili attached with FimH adhesin that facilitates colonization on silicone implants and on surface of urinary bladder leading to CAUTI (Catheter Associated Urinary Tract Infections).^212^ This pathogen after entering the host cells bypasses the host immune system and starts aggregating to form large intracellular bacterial communities (IBC) similar to biofilms.^213-215^ A peptide from the gingival crevicular fluids and saliva, named lactoferrin, inhibits the attachment of *S. mutans* and *Streptococcus gordonii* and prevents the formation of biofilm in oral cavity.^216^ It is also evident from other studies that the presence of lactoferrin prevents the biofilm formation by *Porphyromonas gingivalis* and *Prevotella intermedia* in the subgingival plaque at a very low concentration of ≥ 8 μg/mL.^217^ Mannosides are small molecules that work as an inhibitor of FimH by blocking their functions.^218-220^ Murine model was used to investigate the effect of mannosides for treating CAUTI and it was found that the compound effectively inhibited invasion and colonization on the urinary bladder epithelium after infection by UPEC of the implanted bladders. It also enhanced the activity of trimethoprim-sulfamethoxazole when administered orally for the treatment of urinary tract infections.^66^ Additionally, mannosides inhibited formation of biofilm on silicone surface in-vitro and if it is used for treatment in humans, it resulted in the reduction of CAUTI rates via inhibiting colonization and invasion of UPEC in bladder and also by not letting to form biofilm on the catheter surface.^221^ Another study shows that pilicides, inhibitor of Chaperone/Usher Pathway pili inhibit type I piliation and dysregulates virulence factors of UPEC thus affecting its growth.^222^ Both these molecules are of much clinical relevance and if used to treat UTI or CAUTI current guidelines must be followed. Mannosides may also be used along with other potential antibacterial agents or other preventive compounds before inserting catheter in patient's body as a preventive measure.^223^ In addition to these, a plant derived compound, eugenol is suggested to inhibit early biofilm formation and also reduce preformed biofilm of *S. mutans*. It does not affect the bacterial viability, but downregulates the expression of virulence genes involved in the adhesion and formation of biofilm such as comDE, ftf, smu630, vicR, gtfB, relA, gbpB, gtfC, brpA, and spaPat sub-MIC level.^141^

## Inhibition of biofilm by polysaccharides

**Figure 9.**
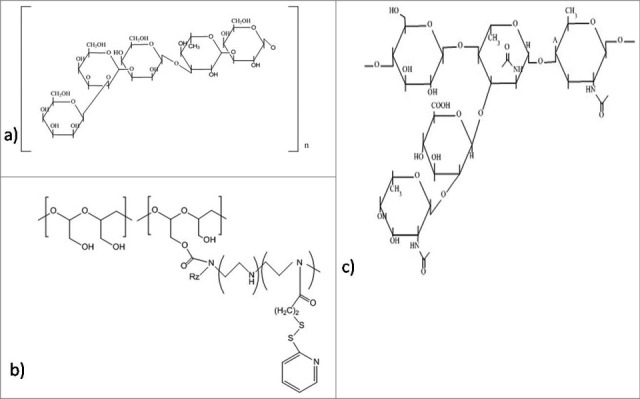
Structures of the anti-biofilm molecules that inhibit polysaccharides. (a) Psl polysaccharide^292,293^, (b) Pel polysaccharide^294^, (c) CFT073 group-II capsular polysaccharide (Serotype K2)^295^.

Extracellular polysachharides is an essential component of biofilms. Recently, a few exo-polysaccharides have been found to show negative activity against biofilm formation. They not only inhibit the biofilm formation^224,225^ but can also lead to the dispersion of the preformed biofilm. A recent experiment have reported an exo-polysaccharide EPS273, obtained from a marine bacterium, *P. stutzeri* 273 that reduces biofilm formation in *P. aeruginosa* by targeting virulence factors which include exoprotease, pyocyanin, and rhamnose. EPS273 interferes with pyocyanin production whose reduction causes the decrease in the production of H_2_O_2_ ultimately inhibiting the release of eDNA which is required for the formation of stable biofilms.^226^ It is also reported that this molecule reduces biofilm related infection in lung cells and embryos of zebrafish and also acts as a potent antioxidant, thereby decreasing the hydroxy radicals and superoxide radicals. Therefore, EPS273 can find its use in healthcare as well as in food industry against *P. aeruginosa*, which is responsible for causing nosocomial infections and spoilage of food respectably. Structural studies revealed that EPS273 has typical characteristics of polysaccharide. 35.4% glucosamine, 28.6% rhamnose, 27.2% glucose, and 8.7% mannose are the predominant monosaccharide units found in EPS273. Molecular weight of this molecule was reported to be 190kDa via HPGPC analysis.^227^ Other various anti-biofilm polysaccharides have also been reported. Psl and Pel (Fig. 9) from *P. aeruginosa* PAO1 decrease the ability to form biofilm by *S. epidermidis* in dual-species biofilm *in-vitro* conditions.^228,229^ K2 polysaccharide (Fig. 9) from the capsule of *E. coli* and PAM galactan from strains of *K. kingae* regulate their own biofilm architecture like forming water channels or dispersal of the biofilm thereby inhibiting their own biofilm according to their surrounding environment.^230,231^ Another polysaccharide, A101 from *V. cholerae* QY101 causes dispersal of the biofilm formed by *P. aeruginosa*.^224^ An exopolysaccharide, PAM galactan from the biofilm of *K. kingae* has also been reported to disperse biofilm of *S. epidermidis*.^230^ Many non-bacterial polysaccharides extracted from plant, animal and some algae are also reported to possess anti-biofilm activity.^225^ These anti-biofilm polysaccharides, especially the ones that are of bacterial origin portray broad-spectrum anti-biofilm activity while only some are capable of dispersing biofilms in their initial stages before attaining maturity. Different oligosaccharides or polysaccharides exhibiting anti-biofilm properties can be used in industrial and clinical settings which are greatly inhabited by antibiotic resistant biofilms causing variety of nosocomial infections. They can be used as adjuvant with available antibiotics reducing their minimum biofilm eradication concentration,^224^ anti-adhesive coating decreasing chances of infections related to medical devices,^230-233^ and probiotics to deliver saccharide prebiotics.^234^ Another protein PslG produced by *P. aeruginosa*, participates in biosynthesis of Psl, one of the most important polysaccharide of its biofilm matrix. Structural analysis of PslG revealed that it is an endoglycosidase and according to reports endogenous administration of PslG disperses preformed matured biofilm and inhibits biofilm formation by targeting the Psl in the matrix. *Ex-vivo* studies revealed that the treatment with PslG, increases the susceptibility of biofilms toward antimicrobials and host immune system.^235^

## Inhibition of c-di-GMP signaling system

Bacteria exist in 3 distinct forms namely; planktonic state responsible for acute infections and can be easily eradicated by administration of required dosage of antibiotics, biofilm state responsible for chronic infections and difficult to treat with antibiotics. The third form is the dispersed state, a distinct stage during transition between biofilm and planktonic state. The process of dispersal facilitates biofilm to spread infections within the host and also result in the transmission of bacteria between different hosts. Cyclic di-GMP (c-di-GMP) is a secondary messenger that play role in the biofilm formation. Modification of signaling pathways of c-di-GMP in bacteria can alter the biofilm formation and its dispersal in clinical environment.^236^ Under stress conditions such as starvation, nitrosative conditions, etc., the bacterial cells lower the amount of c-di-GMP by the activation of phosphodiesterase leading to the dispersal of biofilm. This study also showed that biofilm-dispersed cells are very distinct from biofilm as well as planktonic cells in physiology as well as in their capacity of pathogenesis. Dispersed cells are found to be more virulent against *C. elegans* and immune cells, due to high level expression of virulence related genes as compared with biofilm and planktonic cells. In addition to reduced c-di-GMP concentration, the biofilm-dispersed cells experience a reduced rsmY and rsmZ expression resulting in low siderophore production by bacterial species.^237^ The siderophores function by chelating iron from the environment and are found to be involved in the prevention of biofilm formation by reducing the survival of dispersed cells. On administration of chemicals, the dispersal based anti-biofilm activity gets induced. These dispersed cells can escape the macrophage-mediated phagocytosis, therefore along with the dispersing agents; administration of some antimicrobial agent is preferred, as it would hinder the growth and the spread of dispersed cells. The addition of an iron chelator with the dispersing agent and antimicrobial agent would possibly eradicate the biofilm.^237^

LP 3134, LP 3145, LP 4010 and LP 1062 are the small molecules that inhibit diguanylate cyclase (DGC) that mediates the synthesis of c-di-GMP and hence inhibit biofilm formation in *P. aeruginosa* and *Acinetobacter baumannii*. All of these molecules have been reported to inhibit the biofilm dispersal of *P. aeruginosa*. Among these, only 2 were potential candidates for inhibiting biofilms because they were non-toxic to eukaryotic cells.^6,238^

Some other molecules were also identified as inhibitors of the allosteric binding of c-di-GMP, from studies subjected to differential radial capillary action of ligand assay. DGC activity was reduced by the administration of a synthetic organoselenium drug, ebselen, and binding of c-di-GMP was inhibited by ebselen oxide. Therefore, these 2 molcules can regulate the production of biofilm in *P. aeruginosa*.^239^ Indole signaling is considered as one of the most important signaling pathway that is responsible for various pathogenicity related bacterial behaviors such as virulence,^240^ acid tolerance,^241^ biofilm formation,^242^ resistance to antibiotics.^243^ Studies performed by Bunders et al. revealed that derivatives of desformylflustra bromine (dFBr) result in the inhibition of production of biofilm via modulating the signaling pathway of indole in *S. aureus and E. coli.*^244^

## Molecules with unknown mechanism

**Figure 10.**
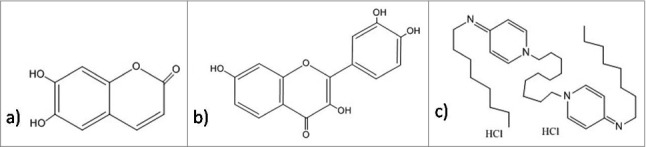
Structures of the antibiofilm molecules with unknown mechanism of action. (a) Esculetin^296^, b) Fisetin^297^, c) Octenidine hydrochloride^298^.

Some of the antibiofilm molecules are reported to work very efficiently but their mechanism of action is yet to be discovered. Secondary metabolites fisetin (Fig. 10) and esculetin (Fig. 10) are known to inhibit biofilm. Esculetin treatment affects the structural maturation of biofilm thereby, reducing its thickness. In contrast to this, fisetin not only reduces thickness of mature biofilm but also, interferes with the initiation of biofilm formation, reducing the coverage area. Hence, fisetin is considered better antibiofilm agent than esculetin.^245^ A positively charged bispyridinamine, octenidine hydrochloride (Fig. 10) is also suggested to be an effective anti-biofilm agent but its mode of action is still unclear. Studies demonstrate that this compound can be potentially used as sanitizer and antimicrobial lock solution in both treatment and prophylactic activities.^246^

## Cytotoxicity of Anti-Biofilm molecules

Cytotoxicity is the most important factor for assessing any adverse effect of the anti-biofilm molecules before using them commercially for the prevention and removal of biofilm. Various methods are there for testing cytotoxic effect namely Lactate dehydrogenase (LDH) assay, MTT assay, XTT assay, Trypan blue, crystal violet, colony formation method, DAPI and PI. Naturally derived compounds from plants are usually not toxic. Cytotoxicity studies have been performed in a variety of species, and the results demonstrate that octenidine hydrochloride is not absorbed through the gastrointestinal tract and, mucous membrane with no reported genotoxicity, carcinogenicity, or mutagenicity.^247^ Likewise, it has been observed that usnic acid might show little side effects such as allergic contact dermatitis and local irritation. *In-vitro* studies revealed no cytotoxic effects of this compound when tested alone or as a constituent of oral formulation. Moreover no toxic effects were evident in pharmacokinetic studies as well as after oral administration.^132,175^ Studies also suggested that AMPs do not show any cytotoxic effects.^248^ Similarly, many antibiofilm compounds like S-phenyl-L-cysteine sulfoxide and its derivative diphenyl disulfide (inhibits biofilm via quorum sensing inhibition) was reported to be non-toxic and non-lethal when tested in drosophila-based infection models.^249^ Apart from testing cytotoxicity of anti-biofilm molecules, some other studies are also required such as permeability studies, plasma protein binding, efflux studies and solubility (in water and salt) studies.

## Conclusions and future prospective

Till date, a lot has already been studied and understood about the bacterial biofilm formation. Emergence of severe biofilm infections and its resistance to antimicrobial treatment, has posed a great challenge in the medical field. The occurrence of resistant bacteria is mainly seen in human, farm animals, fruits, vegetables, dairy products, sea-foods and poultry products. Hence it is essential to investigate the effective ways to combat this problem and find an alternative among antibiotics. To make through this great challenge of today, biofilm imaging techniques have immensely developed. Fluorescence photo-activated localization microscopy (FPLAM), photo-activated localization microscopy (PLAM) and stochastic optical reconstruction microscopy (STORM)^250-252^ are the techniques which involves super-resolution microscopy. They use fluorescent proteins or probes to produce images with much higher resolution than CSLM. Hence, these techniques can be used more often for studying biofilm. To find a significant and effective alternative against biofilm, focus on the discovery of different anti-biofilm molecules along with modifications in the different signaling pathways associated with quorum sensing is taken under consideration. The higher eukaryotes are devoid of cyclic-di-GMP (c-di-GMP) signaling pathway, which makes it an attractive target for designing naive anti-biofilm agents. Besides these, the role of amyloids in bacterial biofilms has also become prevalent. Targeting these amyloids lessens the adherence property of bacterial cells hence affecting the formation of biofilms.^6^ The present study provides the information of the mechanism of action used by different small molecules with anti-biofilm properties. Though, every anti-biofilm molecules have their specific modes of action, but a single molecule may follow more than one mechanism; for example, EGCG works either by inhibiting the AHL-mediated quorum sensing pathway or by degradation of peptidoglycan and membrane disruption. Information regarding mechanism of action provides better understanding about the nature of biofilms, which can be further used to develop new and successful drug molecules with the previously known target of action. This can bring about improvement in the efficacy of the previously known drugs. It can be achieved either by making suitable modifications or by using combinatorial therapy which includes the previously reported less effective drug against bacterial infections along with the potent anti-biofilm agents thereby raising the activity of the antibiotics. According to the present review, EPS273, mannosides and pilicides are the most significant anti-biofilm agents with high clinical relevance but needs further *in-vivo* testing followed by clinical trials. The naturally derived antimicrobials have more biochemical and structural diversity compared with synthetic drugs, thus could be very useful in developing various alternative therapies and *in-silico* pharmaceutical approaches. Additionally, the high complexity of natural products allows enhanced selective binding to the target. The disadvantage of using naturally derived anti-biofilm agents is their high cost, less sustainability; more time consumption and sometimes they show different results once extracted from their sources. In contrary, synthetic drugs are cost effective and consume less time but many of them show adverse side effects. Moreover numerous peptides and polysaccharides due to their large size become inappropriate from a drug perspective. While designing any compound for combating biofilm, it is very important to observe its proper delivery in any *in-vivo* system at a particular site and the dosage of the anti-biofilm compound is another crucial factor, which needs thorough investigation. However, the discovery of new drugs against new targets is likely to be a prolonged and disconcerting process. Therefore, remodeling the previously used drugs approved against target other than biofilms may prove to be a more appropriate and constructive idea. A wide range of bioinformatic tools are available that might be used for the purpose of screening libraries of the existing drugs and to revamp them as a worthwhile contribution. Further, research has to be performed to find out the small active fragments of these peptides and polysaccharides that can effectively bind to the new identified targets.

## References

[1] Alhede M, Kragh KN, Qvortrup K, Allesen-Holm M, van Gennip M, Christensen LD, Jensen PØ, Nielsen AK, Parsek M, Wozniak D, et al. Phenotypes of non-attached Pseudomonas aeruginosa aggregates resemble surface attached biofilm. PloS One 2011; 6:e27943; PMID:22132176; https://doi.org/10.1371/journal.pone.0027943 22132176 PMC3221681

[2] Bjarnsholt T, Jensen PO, Fiandaca MJ, Pedersen J, Hansen CR, Andersen CB, Pressler T, Givskov M, Høiby N Pseudomonas aeruginosa biofilms in the respiratory tract of cystic fibrosis patients. Pediatr Pulmonol 2009; 44:547-58; PMID:19418571; https://doi.org/10.1002/ppul.21011 19418571

[3] Haaber J, Cohn MT, Frees D, Andersen TJ, Ingmer H Planktonic aggregates of Staphylococcus aureus protect against common antibiotics. PloS One 2012; 7:e41075; PMID:22815921; https://doi.org/10.1371/journal.pone.0041075 22815921 PMC3399816

[4] Davies D Understanding biofilm resistance to antibacterial agents. Nat Rev Drug Discov 2003; 2:114-22; PMID:12563302; https://doi.org/10.1038/nrd1008 12563302

[5] Singh PK, Schaefer AL, Parsek MR, Moninger TO, Welsh MJ, Greenberg EP Quorum-sensing signals indicate that cystic fibrosis lungs are infected with bacterial biofilms. Nature 2000; 407:762-4; PMID:11048725; https://doi.org/10.1038/35037627 11048725

[6] Wu H, Moser C, Wang HZ, Hoiby N, Song ZJ Strategies for combating bacterial biofilm infections. Int J Oral Sci 2015; 7:1-7; PMID:25504208; https://doi.org/10.1038/ijos.2014.65 25504208 PMC4817533

[7] Piozzi A, Francolini I, Occhiaperti L, Di Rosa R, Ruggeri V, Donelli G Polyurethanes loaded with antibiotics: influence of polymer-antibiotic interactions on in vitro activity against Staphylococcus epidermidis. J Chemotherapy 2004; 16:446-52; PMID:15565910; https://doi.org/10.1179/joc.2004.16.5.446 15565910

[8] Donelli G, Francolini I Efficacy of antiadhesive, antibiotic and antiseptic coatings in preventing catheter-related infections: review. J Chemotherapy 2001; 13:595-606; PMID:11806619; https://doi.org/10.1179/joc.2001.13.6.595 11806619

[9] Hengzhuang W, Wu H, Ciofu O, Song Z, Hoiby N Pharmacokinetics/pharmacodynamics of colistin and imipenem on mucoid and nonmucoid Pseudomonas aeruginosa biofilms. Antimicrobial Agents Chemotherapy 2011; 55:4469-74; PMID:21670181; https://doi.org/10.1128/AAC.00126-11 21670181 PMC3165294

[10] Hengzhuang W, Wu H, Ciofu O, Song Z, Hoiby N In vivo pharmacokinetics/pharmacodynamics of colistin and imipenem in Pseudomonas aeruginosa biofilm infection. Antimicrobial Agents Chemotherapy 2012; 56:2683-90; PMID:22354300; https://doi.org/10.1128/AAC.06486-11 22354300 PMC3346607

[11] Hoiby N, Ciofu O, Johansen HK, Song ZJ, Moser C, Jensen PO, Molin S, Givskov M, Tolker-Nielsen T, Bjarnsholt T The clinical impact of bacterial biofilms. Int J Oral Sci 2011; 3:55-65; PMID:21485309; https://doi.org/10.4248/IJOS11026 21485309 PMC3469878

[12] Cramton SE, Gerke C, Schnell NF, Nichols WW, Gotz F The intercellular adhesion (ica) locus is present in Staphylococcus aureus and is required for biofilm formation. Infect Immunity 1999; 67:5427-33; PMID:10496925 10496925 10.1128/iai.67.10.5427-5433.1999 PMC96900

[13] Gotz F Staphylococcus and biofilms. Mol Microbiol 2002; 43:1367-78; PMID:11952892; https://doi.org/10.1046/j.1365-2958.2002.02827.x 11952892

[14] McKenney D, Hubner J, Muller E, Wang Y, Goldmann DA, Pier GB The ica locus of Staphylococcus epidermidis encodes production of the capsular polysaccharide/adhesin. Infect Immunity 1998; 66:4711-20; PMID:9746568 9746568 10.1128/iai.66.10.4711-4720.1998 PMC108579

[15] Aaron SD, Ferris W, Ramotar K, Vandemheen K, Chan F, Saginur R Single and combination antibiotic susceptibilities of planktonic, adherent, and biofilm-grown Pseudomonas aeruginosa isolates cultured from sputa of adults with cystic fibrosis. J Clin Microbiol 2002; 40:4172-9; PMID:12409393; https://doi.org/10.1128/JCM.40.11.4172-4179.2002 12409393 PMC139693

[16] Brandl K, Plitas G, Mihu CN, Ubeda C, Jia T, Fleisher M, Schnabl B, DeMatteo RP, Pamer EG Vancomycin-resistant enterococci exploit antibiotic-induced innate immune deficits. Nature 2008; 455:804-7; PMID:18724361; https://doi.org/10.1038/nature07250 18724361 PMC2663337

[17] Hoyle BD, Costerton JW Bacterial resistance to antibiotics: the role of biofilms. Progress Drug Res Fortschritte Der Arzneimittelforschung Progres Des Recherches Pharmaceutiques 1991; 37:91-105; PMID:1763187 1763187 10.1007/978-3-0348-7139-6_2

[18] Moreau-Marquis S, Stanton BA, O'Toole GA Pseudomonas aeruginosa biofilm formation in the cystic fibrosis airway. Pulmonary Pharmacol Therapeutics 2008; 21:595-9; https://doi.org/10.1016/j.pupt.2007.12.001 PMC2542406 18234534

[19] Parsek MR, Singh PK Bacterial biofilms: an emerging link to disease pathogenesis. Annual Rev Microbiol 2003; 57:677-701; PMID:14527295; https://doi.org/10.1146/annurev.micro.57.030502.090720 14527295

[20] Rasmussen TB, Givskov M Quorum sensing inhibitors: a bargain of effects. Microbiol 2006; 152:895-904; PMID:16549654; https://doi.org/10.1099/mic.0.28601-0 16549654

[21] Ciofu O, Mandsberg LF, Wang H, Hoiby N Phenotypes selected during chronic lung infection in cystic fibrosis patients: implications for the treatment of Pseudomonas aeruginosa biofilm infections. FEMS Immunol Medical Microbiol 2012; 65:215-25; PMID:22540844; https://doi.org/10.1111/j.1574-695X.2012.00983.x 22540844

[22] Anderl JN, Zahller J, Roe F, Stewart PS Role of nutrient limitation and stationary-phase existence in Klebsiella pneumoniae biofilm resistance to ampicillin and ciprofloxacin. Antimicrobial Agents Chemotherapy 2003; 47:1251-6; PMID:12654654; https://doi.org/10.1128/AAC.47.4.1251-1256.2003 12654654 PMC152508

[23] Brown MR, Allison DG, Gilbert P Resistance of bacterial biofilms to antibiotics: a growth-rate related effect? J Antimicrobial Chemotherapy 1988; 22:777-80; PMID:3072331; https://doi.org/10.1093/jac/22.6.777 3072331

[24] Walters MC 3rd, Roe F, Bugnicourt A, Franklin MJ, Stewart PS Contributions of antibiotic penetration, oxygen limitation, and low metabolic activity to tolerance of Pseudomonas aeruginosa biofilms to ciprofloxacin and tobramycin. Antimicrobial Agents Chemotherapy 2003; 47:317-23; PMID:12499208; https://doi.org/10.1128/AAC.47.1.317-323.2003 12499208 PMC148957

[25] Ma H, Bryers JD Non-invasive determination of conjugative transfer of plasmids bearing antibiotic-resistance genes in biofilm-bound bacteria: effects of substrate loading and antibiotic selection. Applied Microbiol Biotechnol 2013; 97:317-28; PMID:22669634; https://doi.org/10.1007/s00253-012-4179-9 22669634 PMC3465625

[26] Gjodsbol K, Christensen JJ, Karlsmark T, Jorgensen B, Klein BM, Krogfelt KA Multiple bacterial species reside in chronic wounds: a longitudinal study. Int Wound J 2006; 3:225-31; PMID:16984578; https://doi.org/10.1111/j.1742-481X.2006.00159.x 16984578 PMC7951738

[27] Kirketerp-Moller K, Jensen PO, Fazli M, Madsen KG, Pedersen J, Moser C, Tolker-Nielsen T, Høiby N, Givskov M, Bjarnsholt T Distribution, organization, and ecology of bacteria in chronic wounds. J Clin Microbiol 2008; 46:2717-22; PMID:18508940; https://doi.org/10.1128/JCM.00501-08 18508940 PMC2519454

[28] Homoe P, Bjarnsholt T, Wessman M, Sorensen HC, Johansen HK Morphological evidence of biofilm formation in Greenlanders with chronic suppurative otitis media. Eur Arch Oto-Rhino-Laryngol 2009; 266:1533-8; https://doi.org/10.1007/s00405-009-0940-9 19283404

[29] Boles BR, Horswill AR Agr-mediated dispersal of Staphylococcus aureus biofilms. PLoS Pathogens 2008; 4:e1000052; PMID:18437240; https://doi.org/10.1371/journal.ppat.1000052 18437240 PMC2329812

[30] Van Oss CJ, Good RJ, Chaudhury MK The role of van der Waals forces and hydrogen bonds in “hydrophobic interactions” between biopolymers and low energy surfaces. J Colloid Interface Sci 1986; 111:378-90; https://doi.org/10.1016/0021-9797(86)90041-X

[31] O'Neill E, Pozzi C, Houston P, Humphreys H, Robinson DA, Loughman A, Foster TJ, O'Gara JP A novel Staphylococcus aureus biofilm phenotype mediated by the fibronectin-binding proteins, FnBPA and FnBPB. J Bacteriol 2008; 190:3835-50; PMID:18375547; https://doi.org/10.1128/JB.00167-08 18375547 PMC2395027

[32] Merino N, Toledo-Arana A, Vergara-Irigaray M, Valle J, Solano C, Calvo E, Lopez JA, Foster TJ, Penadés JR, Lasa I Protein A-mediated multicellular behavior in Staphylococcus aureus. J Bacteriol 2009; 191:832-43; PMID:19047354; https://doi.org/10.1128/JB.01222-08 19047354 PMC2632097

[33] Corrigan RM, Rigby D, Handley P, Foster TJ The role of Staphylococcus aureus surface protein SasG in adherence and biofilm formation. Microbiol 2007; 153:2435-46; PMID:17660408; https://doi.org/10.1099/mic.0.2007/006676-0 17660408

[34] Conrady DG, Brescia CC, Horii K, Weiss AA, Hassett DJ, Herr AB A zinc-dependent adhesion module is responsible for intercellular adhesion in staphylococcal biofilms. Proc Natl Acad Sci U S A 2008; 105:19456-61; PMID:19047636; https://doi.org/10.1073/pnas.0807717105 19047636 PMC2592360

[35] Marti M, Trotonda MP, Tormo-Mas MA, Vergara-Irigaray M, Cheung AL, Lasa I, Penadés JR Extracellular proteases inhibit protein-dependent biofilm formation in Staphylococcus aureus. Microbes Infect / Institut Pasteur 2010; 12:55-64; https://doi.org/10.1016/j.micinf.2009.10.005 19883788

[36] Trotonda MP, Manna AC, Cheung AL, Lasa I, Penades JR SarA positively controls bap-dependent biofilm formation in Staphylococcus aureus. J Bacteriol 2005; 187:5790-8; PMID:16077127; https://doi.org/10.1128/JB.187.16.5790-5798.2005 16077127 PMC1196089

[37] Fuqua WC, Winans SC, Greenberg EP Quorum sensing in bacteria: the LuxR-LuxI family of cell density-responsive transcriptional regulators. J Bacteriol 1994; 176:269-75; PMID:8288518; https://doi.org/10.1128/jb.176.2.269-275.1994 8288518 PMC205046

[38] Huber B, Eberl L, Feucht W, Polster J Influence of polyphenols on bacterial biofilm formation and quorum-sensing. Zeitschrift fur Naturforschung C. J Biosci 2003; 58:879-84; PMID:14713169 14713169 10.1515/znc-2003-11-1224

[39] Overhage J, Campisano A, Bains M, Torfs EC, Rehm BH, Hancock RE Human host defense peptide LL-37 prevents bacterial biofilm formation. Infect Immunity 2008; 76:4176-82; PMID:18591225; https://doi.org/10.1128/IAI.00318-08 18591225 PMC2519444

[40] Anderl JN, Franklin MJ, Stewart PS Role of antibiotic penetration limitation in Klebsiella pneumoniae biofilm resistance to ampicillin and ciprofloxacin. Antimicrobial Agents Chemotherapy 2000; 44:1818-24; PMID:10858336; https://doi.org/10.1128/AAC.44.7.1818-1824.2000 10858336 PMC89967

[41] Lappin-Scott HM, Costerton JW Bacterial biofilms and surface fouling. Biofouling 1989; 1:323-42; https://doi.org/10.1080/08927018909378120

[42] Gjermansen M, Nilsson M, Yang L, Tolker-Nielsen T Characterization of starvation-induced dispersion in Pseudomonas putida biofilms: genetic elements and molecular mechanisms. Mol Microbiol 2010; 75:815-26; PMID:19602146; https://doi.org/10.1111/j.1365-2958.2009.06793.x 19602146

[43] Gjermansen M, Ragas P, Sternberg C, Molin S, Tolker-Nielsen T Characterization of starvation-induced dispersion in Pseudomonas putida biofilms. Environmental Microbiol 2005; 7:894-906; PMID:15892708; https://doi.org/10.1111/j.1462-2920.2005.00775.x 15892708

[44] Nilsson M, Chiang WC, Fazli M, Gjermansen M, Givskov M, Tolker-Nielsen T Influence of putative exopolysaccharide genes on Pseudomonas putida KT2440 biofilm stability. Environmental Microbiol 2011; 13:1357-69; PMID:21507178; https://doi.org/10.1111/j.1462-2920.2011.02447.x 21507178

[45] Jackson KD, Starkey M, Kremer S, Parsek MR, Wozniak DJ Identification of psl, a locus encoding a potential exopolysaccharide that is essential for Pseudomonas aeruginosa PAO1 biofilm formation. J Bacteriol 2004; 186:4466-75; PMID:15231778; https://doi.org/10.1128/JB.186.14.4466-4475.2004 15231778 PMC438565

[46] Matsukawa M, Greenberg EP Putative exopolysaccharide synthesis genes influence Pseudomonas aeruginosa biofilm development. J Bacteriol 2004; 186:4449-56; PMID:15231776; https://doi.org/10.1128/JB.186.14.4449-4456.2004 15231776 PMC438629

[47] Wozniak DJ, Wyckoff TJ, Starkey M, Keyser R, Azadi P, O'Toole GA, Parsek MR Alginate is not a significant component of the extracellular polysaccharide matrix of PA14 and PAO1 Pseudomonas aeruginosa biofilms. Proc Natl Acad Sci U S A 2003; 100:7907-12; PMID:12810959; https://doi.org/10.1073/pnas.1231792100 12810959 PMC164686

[48] Costerton JW, Cheng KJ, Geesey GG, Ladd TI, Nickel JC, Dasgupta M, Marrie TJ Bacterial biofilms in nature and disease. Ann Rev Microbiol 1987; 41:435-64; PMID:3318676; https://doi.org/10.1146/annurev.mi.41.100187.002251 3318676

[49] Purevdorj-Gage B, Costerton WJ, Stoodley P Phenotypic differentiation and seeding dispersal in non-mucoid and mucoid Pseudomonas aeruginosa biofilms. Microbiology 2005; 151:1569-76; PMID:15870466; https://doi.org/10.1099/mic.0.27536-0 15870466

[50] Chen Y, Chai Y, Guo JH, Losick R Evidence for cyclic Di-GMP-mediated signaling in Bacillus subtilis. J Bacteriol 2012; 194:5080-90; PMID:22821967; https://doi.org/10.1128/JB.01092-12 22821967 PMC3430322

[51] Garcia B, Latasa C, Solano C, Garcia-del Portillo F, Gamazo C, Lasa I Role of the GGDEF protein family in Salmonella cellulose biosynthesis and biofilm formation. Mol Microbiol 2004; 54:264-77; PMID:15458421; https://doi.org/10.1111/j.1365-2958.2004.04269.x 15458421

[52] Gjermansen M, Ragas P, Tolker-Nielsen T Proteins with GGDEF and EAL domains regulate Pseudomonas putida biofilm formation and dispersal. FEMS Microbiol Letters 2006; 265:215-24; PMID:17054717; https://doi.org/10.1111/j.1574-6968.2006.00493.x 17054717

[53] Hickman JW, Tifrea DF, Harwood CS A chemosensory system that regulates biofilm formation through modulation of cyclic diguanylate levels. Proc Natl Acad Sci U S A 2005; 102:14422-7; PMID:16186483; https://doi.org/10.1073/pnas.0507170102 16186483 PMC1234902

[54] Purcell EB, McKee RW, McBride SM, Waters CM, Tamayo R Cyclic diguanylate inversely regulates motility and aggregation in Clostridium difficile. J Bacteriol 2012; 194:3307-16; PMID:22522894; https://doi.org/10.1128/JB.00100-12 22522894 PMC3434733

[55] Ross P, Weinhouse H, Aloni Y, Michaeli D, Weinberger-Ohana P, Mayer R, Braun S, de Vroom E, van der Marel GA, van Boom JH, et al. Regulation of cellulose synthesis in Acetobacter xylinum by cyclic diguanylic acid. Nature 1987; 325:279-81; PMID:18990795; https://doi.org/10.1038/325279a0 18990795

[56] Ryan RP, Lucey J, O'Donovan K, McCarthy Y, Yang L, Tolker-Nielsen T, Dow JM HD-GYP domain proteins regulate biofilm formation and virulence in Pseudomonas aeruginosa. Environ Microbiol 2009; 11:1126-36; PMID:19170727; https://doi.org/10.1111/j.1462-2920.2008.01842.x 19170727

[57] Simm R, Morr M, Kader A, Nimtz M, Romling U GGDEF and EAL domains inversely regulate cyclic di-GMP levels and transition from sessility to motility. Mol Microbiol 2004; 53:1123-34; PMID:15306016; https://doi.org/10.1111/j.1365-2958.2004.04206.x 15306016

[58] Tischler AD, Camilli A Cyclic diguanylate (c-di-GMP) regulates Vibrio cholerae biofilm formation. Mol Microbiol 2004; 53:857-69; PMID:15255898; https://doi.org/10.1111/j.1365-2958.2004.04155.x 15255898 PMC2790424

[59] Kulasakara H, Lee V, Brencic A, Liberati N, Urbach J, Miyata S, Lee DG, Neely AN, Hyodo M, Hayakawa Y, et al. Analysis of Pseudomonas aeruginosa diguanylate cyclases and phosphodiesterases reveals a role for bis-(3′-5′)-cyclic-GMP in virulence. Proc Natl Acad Sci U S A 2006; 103:2839-44; PMID:16477007; https://doi.org/10.1073/pnas.0511090103 16477007 PMC1413825

[60] Lim B, Beyhan S, Meir J, Yildiz FH Cyclic-diGMP signal transduction systems in Vibrio cholerae: modulation of rugosity and biofilm formation. Mol Microbiol 2006; 60:331-48; PMID:16573684; https://doi.org/10.1111/j.1365-2958.2006.05106.x 16573684

[61] Ryan RP, Tolker-Nielsen T, Dow JM When the PilZ don't work: effectors for cyclic di-GMP action in bacteria. Trends Microbiol 2012; 20:235-42; PMID:22444828; https://doi.org/10.1016/j.tim.2012.02.008 22444828

[62] Tischler AD, Camilli A Cyclic diguanylate regulates Vibrio cholerae virulence gene expression. Infect Immunity 2005; 73:5873-82; PMID:16113306; https://doi.org/10.1128/IAI.73.9.5873-5882.2005 16113306 PMC1231145

[63] Wilksch JJ, Yang J, Clements A, Gabbe JL, Short KR, Cao H, Cavaliere R, James CE, Whitchurch CB, Schembri MA, et al. MrkH, a novel c-di-GMP-dependent transcriptional activator, controls Klebsiella pneumoniae biofilm formation by regulating type 3 fimbriae expression. PLoS Pathogens 2011; 7:e1002204; PMID:21901098; https://doi.org/10.1371/journal.ppat.1002204 21901098 PMC3161979

[64] Massie JP, Reynolds EL, Koestler BJ, Cong JP, Agostoni M, Waters CM Quantification of high-specificity cyclic diguanylate signaling. Proc Natl Acad Sci U S A 2012; 109:12746-51; PMID:22802636; https://doi.org/10.1073/pnas.1115663109 22802636 PMC3411991

[65] Monds RD, Newell PD, Gross RH, O'Toole GA Phosphate-dependent modulation of c-di-GMP levels regulates Pseudomonas fluorescens Pf0-1 biofilm formation by controlling secretion of the adhesin LapA. Mol Microbiol 2007; 63:656-79; PMID:17302799; https://doi.org/10.1111/j.1365-2958.2006.05539.x 17302799

[66] O'Connor JR, Kuwada NJ, Huangyutitham V, Wiggins PA, Harwood CS Surface sensing and lateral subcellular localization of WspA, the receptor in a chemosensory-like system leading to c-di-GMP production. Mol Microbiol 2012; 86:720-9; PMID:22957788; https://doi.org/10.1111/mmi.12013 22957788 PMC3501340

[67] Borlee BR, Goldman AD, Murakami K, Samudrala R, Wozniak DJ, Parsek MR Pseudomonas aeruginosa uses a cyclic-di-GMP-regulated adhesin to reinforce the biofilm extracellular matrix. Mol Microbiol 2010; 75:827-42; PMID:20088866; https://doi.org/10.1111/j.1365-2958.2009.06991.x 20088866 PMC2847200

[68] Chambers JR, Sauer K Small RNAs and their role in biofilm formation. Trends Microbiol 2013; 21:39-49; PMID:23178000; https://doi.org/10.1016/j.tim.2012.10.008 23178000 PMC3752386

[69] Antonelli M, De Pascale G, Ranieri VM, Pelaia P, Tufano R, Piazza O, Zangrillo A, Ferrario A, De Gaetano A, Guaglianone E, et al. Comparison of triple-lumen central venous catheters impregnated with silver nanoparticles (AgTive(R)) vs conventional catheters in intensive care unit patients. J Hospital Infect 2012; 82:101-7; PMID:22938728; https://doi.org/10.1016/j.jhin.2012.07.010 22938728

[70] Heersink J, Goeres D Reactor design considerations. In: Hamilton M, Heersink J, Buckingham-Meyer K, Goeres D, editors. The Biofilm Laboratory: Step-by-step Protocols for Experimental Design, Analysis, and Data Interpretation, Hamilton M., Heersink J., Buckingham-Meyer K. and Goeres D., eds. Bozeman, MT: Cytergy Publishing 2003:13-5.

[71] Niu C, Gilbert ES Colorimetric method for identifying plant essential oil components that affect biofilm formation and structure. Appl Environ Microbiol 2004; 70:6951-6; PMID:15574886; https://doi.org/10.1128/AEM.70.12.6951-6956.2004 15574886 PMC535164

[72] Heilmann C, Gerke C, Perdreau-Remington F, Gotz F Characterization of Tn917 insertion mutants of Staphylococcus epidermidis affected in biofilm formation. Infect Immunity 1996; 64:277-82; PMID:8557351 8557351 10.1128/iai.64.1.277-282.1996 PMC173756

[73] O'Toole GA, Kolter R Flagellar and twitching motility are necessary for Pseudomonas aeruginosa biofilm development. Mol Microbiol 1998; 30:295-304; PMID:9791175; https://doi.org/10.1046/j.1365-2958.1998.01062.x 9791175

[74] Coenye T, Nelis HJ In vitro and in vivo model systems to study microbial biofilm formation. J Microbiological Methods 2010; 83:89-105; PMID:20816706; https://doi.org/10.1016/j.mimet.2010.08.018 20816706

[75] O'Toole GA Microtiter dish biofilm formation assay. J Vis Exp 2011; 47:2473; PMID:21307833 21307833 10.3791/2437 PMC3182663

[76] Busscher HJ, van der Mei HC Microbial adhesion in flow displacement systems. Clin Microbiol Rev 2006; 19:127-41; PMID:16418527; https://doi.org/10.1128/CMR.19.1.127-141.2006 16418527 PMC1360269

[77] Debebe T, Krüger M, Huse K, Kacza J, Mühlberg K, König B, Birkenmeier G Ethyl Pyruvate: An anti-microbial agent that selectively targets Pathobionts and Biofilms. PLoS One 2016; 11:e0162919; https://doi.org/10.1371/journal.pone.0162919 27658257 PMC5033407

[78] Maske TT, Brauner KV, Nakanishi L, Arthur RA, van de Sande FH, Cenci MS An in vitro dynamic microcosm biofilm model for caries lesion development and antimicrobial dose-response studies. Biofouling 2016; 32:339-48; PMID:26905384; https://doi.org/10.1080/08927014.2015.1130824 26905384

[79] Salli KM, Ouwehand AC The use of in vitro model systems to study dental biofilms associated with caries: a short review. J Oral Microbiol 2015; 7:26149; PMID:25740099; https://doi.org/10.3402/jom.v7.26149 25740099 PMC4349908

[80] Singer G, Besemer K, Hödl I, Chlup A, Hochedlinger G, Stadler P, et al. Microcosm design and evaluation to study stream microbial biofilms. Limnol Oceanogr Methods 2006; 4(11):436-47; https://doi.org/10.4319/lom.2006.4.436

[81] Lebeaux D, Chauhan A, Rendueles O, Beloin C From in vitro to in vivo Models of Bacterial Biofilm-Related Infections. Pathogens 2013; 2:288-356; PMID:25437038; https://doi.org/10.3390/pathogens2020288 25437038 PMC4235718

[82] Lemaitre B, Ausubel FM Animal models for host-pathogen interactions. Curr Opin Microbiol 2008; 11:249-50; PMID:18539076; https://doi.org/10.1016/j.mib.2008.05.002 18539076

[83] Djordjevic D, Wiedmann M, McLandsborough LA Microtiter plate assay for assessment of Listeria monocytogenes biofilm formation. Applied Environ Microbiol 2002; 68:2950-8; PMID:12039754; https://doi.org/10.1128/AEM.68.6.2950-2958.2002 12039754 PMC123944

[84] Hassan A, Usman J, Kaleem F, Omair M, Khalid A, Iqbal M Evaluation of different detection methods of biofilm formation in the clinical isolates. Braz J Infect Dis 2011; 15:305-11; PMID:21860999; https://doi.org/10.1016/S1413-8670(11)70197-0 21860999

[85] Freeman DJ, Falkiner FR, Keane CT New method for detecting slime production by coagulase negative staphylococci. J Clin Pathol 1989; 42:872-4; PMID:2475530; https://doi.org/10.1136/jcp.42.8.872 2475530 PMC1142068

[86] Sánchez MC, Llama-Palacios A, Marín MJ, Figuero E, León R, Blanc V, Herrera D, Sanz M Validation of ATP bioluminescence as a tool to assess antimicrobial effects of mouthrinses in an in vitro subgingival-biofilm model. Med Oral Patol Oral Cir Bucal 2013; 18:e86-92; PMID:23229259; https://doi.org/10.4317/medoral.18376 23229259 PMC3548652

[87] Aparna MS, Yadav S Biofims: microbes and disease. Braz J Infect Dis 2008; 12:526-30; PMID:19287843; https://doi.org/10.1590/S1413-86702008000600016 19287843

[88] Dhale RP, Ghorpade MV, Dharmadhikari CA Comparison of various methods used to detect biofilm production of candida species. J Clin Diagnostic Res 2014; 8:DC18-DC20; PMID:25584219 25584219 10.7860/JCDR/2014/10445.5147 PMC4290237

[89] Zufferey J, Rime B, Francioli P, Bille J Simple method for rapid diagnosis of catheter-associated infection by direct acridine orange staining of catheter tips. J Clin Microbiol 1988; 26:175-7; PMID:2449453 2449453 10.1128/jcm.26.2.175-177.1988 PMC266246

[90] Gomes F, Teixeira P, Cerca N, Azeredo J, Oliveira R Effect of farnesol on structure and composition of Staphylococcus epidermidis biofilm matrix. Curr Microbiol 2011; 63:354-9; PMID:21800262; https://doi.org/10.1007/s00284-011-9984-3 21800262

[91] Quintas V, Prada-López I, Tomás I Analyzing the oral biofilm using fluorescence-based microscopy: what's in a dye? In Microscopy: Adv Scientific Res Education, A. Méndez-Vilas, ed. Badajoz, Spain: Formatex Research Center 2014:226-38.

[92] Wiggli M, Smallcombe A, Bachofen R Reflectance spectroscopy and laser confocal microscopy as tools in an ecophysiological study of microbial mats in an alpine bog pond. J Microbiological Methods 1999; 34:173-82; https://doi.org/10.1016/S0167-7012(98)00085-2

[93] Broschat SL, Loge FJ, Peppin JD, White D, Call DR, Kuhn E Optical reflectance assay for the detection of biofilm formation. J Biomedical Optics 2005; 10:44027; PMID:16178660; https://doi.org/10.1117/1.1953347 16178660

[94] Humbert F, Quilès F In-situ study of early stages of biofilm formation under different environmental stresses by ATR-FTIR spectroscopy. In Science against Microbial Pathogens: Communicating Current Research and Technological Advances, A. Méndez-Vilas, ed. Badajoz, Spain: Formatex Research Center 2011:889-95.

[95] Paquet-Mercier F, Safdar M, Parvinzadeh M, Greener J Emerging Spectral Microscopy Techniques and Applications to Biofilm Detection. Microscopy: Advances in Scientific Research and Education, A. Méndez-Vilas, ed. Badajoz, Spain: Formatex Research Center 2014; 2: 638–49.

[96] Hornemann JA, Codd SL, Fell RJ, Stewart PS, Seymour JD Secondary flow mixing due to biofilm growth in capillaries of varying dimensions. Biotechnol Bioengineering 2009; 103:353-60; PMID:19191352; https://doi.org/10.1002/bit.22248 19191352 PMC2744645

[97] Hornemann JA, Lysova AA, Codd SL, Seymour JD, Busse SC, Stewart PS, Brown JR Biopolymer and water dynamics in microbial biofilm extracellular polymeric substance. Biomacromolecules 2008; 9:2322-8; PMID:18665639; https://doi.org/10.1021/bm800269h 18665639 PMC2577057

[98] Sandt C, Smith-Palmer T, Pink J, Brennan L, Pink D Confocal Raman microspectroscopy as a tool for studying the chemical heterogeneities of biofilms in situ. J Appl Microbiol 2007; 103:1808-20; PMID:17953591; https://doi.org/10.1111/j.1365-2672.2007.03413.x 17953591

[99] Sandt C, Smith-Palmer T, Pink J, Brennan L, Pink D Confocal Raman microspectroscopy as a tool for studying the chemical heterogeneities of biofilms in situ. J Appl Microbiol 2007; 103:1808-20; PMID:17953591; https://doi.org/10.1111/j.1365-2672.2007.03413.x 17953591

[100] da Silva WJ, Seneviratne J, Parahitiyawa N, Rosa EA, Samaranayake LP, Del Bel Cury AA Improvement of XTT assay performance for studies involving Candida albicans biofilms. Brazilian Dental J 2008; 19:364-9; PMID:19180329 19180329 10.1590/s0103-64402008000400014

[101] Kuhn DM, George T, Chandra J, Mukherjee PK, Ghannoum MA Antifungal susceptibility of Candida biofilms: unique efficacy of amphotericin B lipid formulations and echinocandins. Antimicrobial Agents Chemotherapy 2002; 46:1773-80; https://doi.org/10.1128/AAC.46.6.1773-1780.2002 12019089 PMC127206

[102] Piper KE, Jacobson MJ, Cofield RH, Sperling JW, Sanchez-Sotelo J, Osmon DR, McDowell A, Patrick S, Steckelberg JM, Mandrekar JN Microbiologic diagnosis of prosthetic shoulder infection by use of implant sonication. J Clin Microbiol 2009; 47:1878-84; https://doi.org/10.1128/JCM.01686-08 19261785 PMC2691098

[103] Song Z, Borgwardt L, Hoiby N, Wu H, Sorensen TS, Borgwardt A Prosthesis infections after orthopedic joint replacement: the possible role of bacterial biofilms. Orthopedic Rev 2013; 5:65-71; PMID:23888204; https://doi.org/10.4081/or.2013.e14 23888204 PMC3718238

[104] Berbari E, Mabry T, Tsaras G, Spangehl M, Erwin PJ, Murad MH, Steckelberg J, Osmon D Inflammatory blood laboratory levels as markers of prosthetic joint infection: A systematic review and meta-analysis. J Bone Joint Surg Am 2010; 92:2102-9; PMID:20810860; https://doi.org/10.2106/JBJS.I.01199 20810860

[105] Vergidis P, Patel R Novel approaches to the diagnosis, prevention, and treatment of medical device-associated infections. Infect Dis Clinics North Am 2012; 26:173-86; PMID:22284383; https://doi.org/10.1016/j.idc.2011.09.012 22284383 PMC3269005

[106] Amann R, Ludwig W Ribosomal RNA-targeted nucleic acid probes for studies in microbial ecology. FEMS Microbiol Rev 2000; 24:555-65; PMID:11077149; https://doi.org/10.1111/j.1574-6976.2000.tb00557.x 11077149

[107] Brileya KA, Camilleri LB, Fields MW 3D- fluorescence *in situ* hybridisation of intact, anaerobic biofilm. Methods Mol Biol 2014; 1151:189-97; PMID:24838887 24838887 10.1007/978-1-4939-0554-6_13

[108] Cassán FD, Okon Y, Creus CM Handbook for Azospirillum: Technical Issues and Protocols, Creus C.M., ed. Switzerland: Springer International 2015; doi:10.1007/978-3-319-06542-7

[109] DeLong EF, Wickham GS, Pace NR Phylogenetic stains: ribosomal RNA-based probes for the identification of single cells. Science 1989; 243:1360-3; PMID:2466341; https://doi.org/10.1126/science.2466341 2466341

[110] Malic S, Hill KE, Hayes A Detection and identification of specific bacteria in wound biofilms using peptide nucleic acid fluorescent in situ hybridization (PNA FISH). Microbiology 2009; 155:2603-11; PMID:19477903; https://doi.org/10.1099/mic.0.028712-0 19477903

[111] Moller S, Pedersen AR, Poulsen LK, Arvin E, Molin S Activity and three-dimensional distribution of toluene-degrading Pseudomonas putida in a multispecies biofilm assessed by quantitative in situ hybridisatrion and scanning confocal laser microscopy. Applied Environ Microbiol 1996; 62:4632-40; PMID:8953734 8953734 10.1128/aem.62.12.4632-4640.1996 PMC168289

[112] Schramm A, De Beer D, Wagner M, Amann R Identification and activities *in situ* of *Nitrosospira* and *Nitrospira spp*. as dominant populations in a nitrifying fluidized bed reactor. Applied Environ Microbiol 1998; 64:3480-5; PMID:9726900 9726900 10.1128/aem.64.9.3480-3485.1998 PMC106750

[113] Skogman ME, Vuorela PM, Fallarero A Combining biofilm matrix measurements with biomass and viability assays in susceptibility assessments of antimicrobials against Staphylococcus aureus biofilms. J Antibiotics 2012; 65:453-9; PMID:22739537; https://doi.org/10.1038/ja.2012.49 22739537

[114] Hoiby N, Krogh Johansen H, Moser C, Song Z, Ciofu O, Kharazmi A Pseudomonas aeruginosa and the in vitro and in vivo biofilm mode of growth. Microbes Infect / Institut Pasteur 2001; 3:23-35; https://doi.org/10.1016/S1286-4579(00)01349-6 11226851

[115] Herrmann G, Yang L, Wu H, Song Z, Wang H, Hoiby N, Ulrich M, Molin S, Riethmüller J, Döring G Colistin-tobramycin combinations are superior to monotherapy concerning the killing of biofilm Pseudomonas aeruginosa. J Infect Dis 2010; 202:1585-92; PMID:20942647; https://doi.org/10.1086/656788 20942647

[116] Francolini I, Piozzi A, Donelli G Efficacy evaluation of antimicrobial drug-releasing polymer matrices. Methods Mol Biology 2014; 1147:215-25; PMID:24664836 24664836 10.1007/978-1-4939-0467-9_15

[117] Donelli G, Francolini I, Ruggeri V, Guaglianone E, D'Ilario L, Piozzi A Pore formers promoted release of an antifungal drug from functionalized polyurethanes to inhibit Candida colonization. J Appl Microbiol 2006; 100:615-22; PMID:16478501; https://doi.org/10.1111/j.1365-2672.2005.02801.x 16478501

[118] Donelli G, Francolini I, Piozzi A, Di Rosa R, Marconi W New polymer-antibiotic systems to inhibit bacterial biofilm formation: a suitable approach to prevent central venous catheter-associated infections. J Chemotherapy 2002; 14:501-7; PMID:12462430; https://doi.org/10.1179/joc.2002.14.5.501 12462430

[119] Crisante F, Taresco V, Donelli G, Vuotto C, Martinelli A, D'Ilario L, et al. Antioxidant Hydroxytyrosol-Based Polyacrylate with Antimicrobial and Antiadhesive Activity Versus Staphylococcus Epidermidis. Adv Exp Med Biol 2015; 901:25-36; PMID:25384665 26542603 10.1007/5584_2015_5013

[120] Percival SL, Suleman L, Francolini I, Donelli G The effectiveness of photodynamic therapy on planktonic cells and biofilms and its role in wound healing. Future Microbiol 2014; 9:1083-94; PMID:25340837; https://doi.org/10.2217/fmb.14.59 25340837

[121] Donelli G, Francolini I, Romoli D, Guaglianone E, Piozzi A, Ragunath C, Kaplan JB Synergistic activity of dispersin B and cefamandole nafate in inhibition of staphylococcal biofilm growth on polyurethanes. Antimicrobial Agents Chemotherapy 2007; 51:2733-40; PMID:17548491; https://doi.org/10.1128/AAC.01249-06 17548491 PMC1932551

[122] Tiwari V, Roy R, Tiwari M Antimicrobial active herbal compounds against Acinetobacter baumannii and other pathogens. Frontiers Microbiol 2015; 6:618; https://doi.org/10.3389/fmicb.2015.00618 PMC4471432 26150810

[123] Hentzer M, Riedel K, Rasmussen TB, Heydorn A, Anderson JB, Parsck MR, Rice SA, Eberl L, Molin S, Høiby N, et al. Inhibition of quorum sensing in Pseudomonas aeruginosa biofilm bacteria by a halogenated furanone compound. Microbiology 2002; 148:87-102; PMID:11782502; https://doi.org/10.1099/00221287-148-1-87 11782502

[124] Gambello MJ, Iglewski BH Cloning and characterization of the Pseudomonas aeruginosa lasR gene, a transcriptional activator of elastase expression. J Bacteriol 1991; 173:3000-9; PMID:1902216; https://doi.org/10.1128/jb.173.9.3000-3009.1991 1902216 PMC207884

[125] Passador L, Cook JM, Gambello MJ, Rust L, Iglewski BH Expression of Pseudomonas aeruginosa virulence genes requires cell-to-cell communication. Science 1993; 260:1127-30; PMID:8493556; https://doi.org/10.1126/science.8493556 8493556

[126] Givskov M, de Nys R, Manefield M, Gram L, Maximilien R, Eberl L, Molin S, Steinberg PD, Kjelleberg S Eukaryotic interference with homoserine lactone-mediated prokaryotic signalling. J Bacteriol 1996; 178:6618-22; PMID:8932319; https://doi.org/10.1128/jb.178.22.6618-6622.1996 8932319 PMC178549

[127] Manefield M, Nys R, Kumar N, Read R, Givskov M, Steinberg P, Kjelleberg S Evidence that halogenated furanones from Delisea pulchra inhibit acylated homoserine lactone (AHL)-mediated gene expression by displacing the AHL signal from its receptor protein. Microbiology 1999; 145:283-91; PMID:10075410; https://doi.org/10.1099/13500872-145-2-283 10075410

[128] Hentzer M, Wu H, Andersen JB, Riedel K, Rasmussen TB, Bagge N, Kumar N, Schembri MA, Song Z, Kristoffersen P, et al. Attenuation of Pseudomonas aeruginosa virulence by quorum sensing inhibitors. EMBO J 2003; 22:3803-15; PMID:12881415; https://doi.org/10.1093/emboj/cdg366 12881415 PMC169039

[129] Manefield M, Harris L, Rice SA, de Nys R, Kjelleberg S Inhibition of luminescence and virulence in the black tiger prawn (Penaeus monodon) pathogen Vibrio harveyi by intercellular signal antagonists. Applied Environmental Microbiol 2000; 66:2079-84; PMID:10788385; https://doi.org/10.1128/AEM.66.5.2079-2084.2000 10788385 PMC101458

[130] Lee JH, Park JH, Cho HS, Joo SW, Cho MH, Lee J Anti-biofilm activities of quercetin and tannic acid against Staphylococcus aureus. Biofouling 2013; 29:491-9; PMID:23668380; https://doi.org/10.1080/08927014.2013.788692 23668380

[131] Manner S, Skogman M, Goeres D, Vuorela P, Fallarero A Systematic exploration of natural and synthetic flavonoids for the inhibition of Staphylococcus aureus biofilms. Int J Mol Sci 2013; 14:19434-51; PMID:24071942; https://doi.org/10.3390/ijms141019434 24071942 PMC3821565

[132] Francolini I, Norris P, Piozzi A, Donelli G, Stoodley P Usnic acid, a natural antimicrobial agent able to inhibit bacterial biofilm formation on polymer surfaces. Antimicrobial Agents Chemotherapy 2004; 48:4360-5; PMID:15504865; https://doi.org/10.1128/AAC.48.11.4360-4365.2004 15504865 PMC525405

[133] Kali A, Bhuvaneshwar D, Charles PM, Seetha KS Antibacterial synergy of curcumin with antibiotics against biofilm producing clinical bacterial isolates. J Basic Clin Pharmacy 2016; 7:93-6; PMID:27330262; https://doi.org/10.4103/0976-0105.183265 27330262 PMC4910474

[134] Fuente-Núñez C, Reffuveille F, Haney EF, Straus SK, Hancock REW Broad-Spectrum anti-biofilm peptide that targets a cellular stress response. PLoS Pathogens 2014; 10:e1004152 24852171 10.1371/journal.ppat.1004152 PMC4031209

[135] Potrykus K, Cashel M (p)ppGpp: still magical? Annual Rev Microbiol 2008; 62:35-51; PMID:18454629; https://doi.org/10.1146/annurev.micro.62.081307.162903 18454629

[136] Abranches J, Martinez AR, Kajfasz JK, Chavez V, Garsin DA, Lemos JA The molecular alarmone (p)ppGpp mediates stress responses, vancomycin tolerance, and virulence in Enterococcus faecalis. J Bacteriol 2009; 191:2248-56; PMID:19168608; https://doi.org/10.1128/JB.01726-08 19168608 PMC2655485

[137] Paz LEC, Lemos JA, Wickström C, Sedgley CM Role of (p)ppGpp in biofilm formation by Enterococcus faecalis. Applied Environmental Microbiol 2012; 78:1627-30; PMID:22179256; https://doi.org/10.1128/AEM.07036-11 22179256 PMC3294496

[138] Reffuveille F, de la Fuente-Núñez C, Mansour S, Hancock REW A broad-spectrum anti-biofilm peptide enhances antibiotic action against bacterial biofilms. Antimicrob Agents Chemother 2014; 58:5363-71; PMID:24982074; https://doi.org/10.1128/AAC.03163-14 24982074 PMC4135845

[139] De la Fuente-Núñez C, Mansour SC, Wang Z, Jiang L, Breidenstein EBM, Elliott M, Reffuveille F, Speert DP, Reckseidler-Zenteno SL, Shen Y, et al. Anti-biofilm and immunomodulatory activities of peptides that inhibit biofilms formed by pathogens isolated from cystic fibrosis patients. Antibiotics 2014; 3:509-26; PMID:26221537; https://doi.org/10.3390/antibiotics3040509 26221537 PMC4515429

[140] de la Fuente-Nunez C, Korolik V, Bains M, Nguyen U, Breidenstein EB, Horsman S, Lewenza S, Burrows L, Hancock RE Inhibition of bacterial biofilm formation and swarming motility by a small synthetic cationic peptide. Antimicrobial Agents Chemotherapy 2012; 56:2696-704; PMID:22354291; https://doi.org/10.1128/AAC.00064-12 22354291 PMC3346644

[141] Lemos JAC, Brown TA, Burne RA Effects of RelA on key virulence properties of planktonic and biofilm populations of Streptococcus mutans. Infect Immunity 2004; 72:1431-40; PMID:14977948; https://doi.org/10.1128/IAI.72.3.1431-1440.2004 14977948 PMC356000

[142] Stewart PS Prospects for anti-biofilm pharmaceuticals. Pharmaceuticals 2015; 8:504-11; PMID:26343685; https://doi.org/10.3390/ph8030504 26343685 PMC4588180

[143] Kaplan JB Therapeutic potential of biofilm-dispersing enzymes. Int J Artif Organs 2009; 32:533-695; PMID:19856268 19851978 10.1177/039139880903200903

[144] Izano EA, Amarante MA, Kher WB, Kaplan JB Differential roles of poly-N-acetylglucosamine surface polysaccharide and extracellular DNA in Staphylococcus aureus and Staphylococcus epidermidis biofilms. Applied Environmental Microbiol 2008; 74:470-6; PMID:18039822; https://doi.org/10.1128/AEM.02073-07 18039822 PMC2223269

[145] Darouiche RO, Mansouri MD, Gawande PV, Madhyastha S Antimicrobial and antibiofilm efficacy of triclosan and DispersinB combination. J Antimicrobial Chemotherapy 2009; 64:88-93; PMID:19447791; https://doi.org/10.1093/jac/dkp158 19447791

[146] Payne DE, Martin NR, Parzych KR, Rickard AH, Underwood A, Boles BR Tannic acid inhibits Staphylococcus aureus surface colonization in an IsaA-dependent manner. Infect Immunity 2013; 81:496-504; PMID:23208606; https://doi.org/10.1128/IAI.00877-12 23208606 PMC3553799

[147] Stapleton MR, Horsburgh MJ, Hayhurst EJ, Wright L, Jonsson IM, Tarkowski A, Kokai-Kun JF, Mond JJ, Foster SJ Characterization of IsaA and SceD, two putative lytic transglycosylases of Staphylococcus aureus. J Bacteriol 2007; 189:7316-25; PMID:17675373; https://doi.org/10.1128/JB.00734-07 17675373 PMC2168438

[148] Holtje JV, Mirelman D, Sharon N, Schwarz U Novel type of murein transglycosylase in Escherichia coli. J Bacteriol 1975; 124:1067-76. 357 10.1128/jb.124.3.1067-1076.1975 PMC236007

[149] Shah IM, Laaberki MH, Popham DL, Dworkin J A eukaryotic-like Ser/Thr kinase signals bacteria to exit dormancy in response to peptidoglycan fragments. Cell 2008; 135:486-96; PMID:18984160; https://doi.org/10.1016/j.cell.2008.08.039 18984160 PMC2892110

[150] Shen Y, Koller T, Kreikemeyer B, Nelson DC Rapid degradation of Streptococcus pyogenes biofilms by PlyC, a bacteriophage-encoded endolysin. J Antimicrobial Chemotherapy 2013; 68:1818-24; PMID:23557924; https://doi.org/10.1093/jac/dkt104 23557924

[151] Fischetti VA Bacteriophage endolysins: a novel anti-infective to control Gram-positive pathogens. Int J Medical Microbiol 2010; 300:357-62; PMID:20452280; https://doi.org/10.1016/j.ijmm.2010.04.002 20452280 PMC3666336

[152] Hoopes JT, Stark CJ, Kim HA, Sussman DJ, Donovan DM, Nelson DC Use of a bacteriophage lysin, PlyC, as an enzyme disinfectant against Streptococcus equi. Applied Environ Microbiol 2009; 75:1388-94; PMID:19139235; https://doi.org/10.1128/AEM.02195-08 19139235 PMC2648168

[153] Koller T, Nelson D, Nakata M, Kreutzer M, Fischetti VA, Glocker MO, Podbielski A, Kreikemeyer B PlyC, a novel bacteriophage lysin for compartment-dependent proteomics of group A streptococci. Proteomics 2008; 8:140-8; PMID:18095374; https://doi.org/10.1002/pmic.200700001 18095374

[154] McGowan S, Buckle AM, Mitchell MS, Hoopes JT, Gallagher DT, Heselpoth RD, Shen Y, Reboul CF, Law RH, Fischetti VA, et al. X-ray crystal structure of the streptococcal specific phage lysin PlyC. Proc Natl Acad Sci U S A 2012; 109:12752-7; PMID:22807482; https://doi.org/10.1073/pnas.1208424109 22807482 PMC3412044

[155] Nelson D, Loomis L, Fischetti VA Prevention and elimination of upper respiratory colonization of mice by group A streptococci by using a bacteriophage lytic enzyme. Proc Natl Acad Sci U S A 2001; 98:4107-12; https://doi.org/10.1073/pnas.061038398 11259652 PMC31187

[156] Nelson D, Schuch R, Chahales P, Zhu S, Fischetti VA PlyC: a multimeric bacteriophage lysin. Proc Natl Acad Sci U S A 2006; 103:10765-70; PMID:16818874; https://doi.org/10.1073/pnas.0604521103 16818874 PMC1487170

[157] Yoda Y, Hu ZQ, Zhao WH, Shimamura T Different susceptibilities of Staphylococcus and Gram-negative rods to epigallocatechin gallate. J Infect Chemotherapy 2004; 10:55-8; PMID:14991521; https://doi.org/10.1007/s10156-003-0284-0 14991521

[158] Zhao WH, Hu ZQ, Hara Y, Shimamura T Inhibition of penicillinase by epigallocatechin gallate resulting in restoration of antibacterial activity of penicillin against penicillinase-producing Staphylococcus aureus. Antimicrobial Agents Chemotherapy 2002; 46:2266-8; PMID:12069986; https://doi.org/10.1128/AAC.46.7.2266-2268.2002 12069986 PMC127279

[159] Carpentier B, Cerf O Biofilms and their consequences, with particular reference to hygiene in the food industry. J Appl Bacteriol 1993; 75:499-511; PMID:8294303; https://doi.org/10.1111/j.1365-2672.1993.tb01587.x 8294303

[160] Boles BR, Horswill AR Staphylococcal biofilm disassembly. Trends Microbiol 2011; 19:449-55; PMID:21784640; https://doi.org/10.1016/j.tim.2011.06.004 21784640 PMC3164736

[161] Thoendel M, Kavanaugh JS, Flack CE, Horswill AR Peptide signaling in the staphylococci. Chem Rev 2011; 111:117-51; PMID:21174435; https://doi.org/10.1021/cr100370n 21174435 PMC3086461

[162] Beenken KE, Mrak LN, Griffin LM, Zielinska AK, Shaw LN, Rice KC, Horswill AR, Bayles KW, Smeltzer MS Epistatic Relationships between sarA and agr in Staphylococcus aureus Biofilm Formation. PloS One 2010; 5; PMID:20520723; https://doi.org/10.1371/journal.pone.0010790 20520723 PMC2875390

[163] Vuong C, Saenz HL, Gotz F, Otto M Impact of the agr quorum-sensing system on adherence to polystyrene in Staphylococcus aureus. J Infect Dis 2000; 182:1688-93; PMID:11069241; https://doi.org/10.1086/317606 11069241

[164] Lauderdale KJ, Boles BR, Cheung AL, Horswill AR Interconnections between Sigma B, agr, and proteolytic activity in Staphylococcus aureus biofilm maturation. Infect Immunity 2009; 77:1623-35; PMID:19188357; https://doi.org/10.1128/IAI.01036-08 19188357 PMC2663138

[165] Tsang LH, Cassat JE, Shaw LN, Beenken KE, Smeltzer MS Factors contributing to the biofilm-deficient phenotype of Staphylococcus aureus sarA mutants. PloS One 2008; 3:e3361; PMID:18846215; https://doi.org/10.1371/journal.pone.0003361 18846215 PMC2556392

[166] Mann EE, Rice KC, Boles BR, Endres JL, Ranjit D, Chandramohan L, Tsang LH, Smeltzer MS, Horswill AR, Bayles KW Modulation of eDNA release and degradation affects Staphylococcus aureus biofilm maturation. PloS One 2009; 4:e5822; PMID:19513119; https://doi.org/10.1371/journal.pone.0005822 19513119 PMC2688759

[167] Branda SS, Chu F, Kearns DB, Losick R, Kolter R A major protein component of the Bacillus subtilis biofilm matrix. Mol Microbiol 2006; 59:1229-38; PMID:16430696; https://doi.org/10.1111/j.1365-2958.2005.05020.x 16430696

[168] Romero D, Kolter R Will biofilm disassembly agents make it to market? Trends Microbiol 2011; 19:304-6; PMID:21458996; https://doi.org/10.1016/j.tim.2011.03.003 21458996 PMC3750235

[169] Cegelski L, Pinkner JS, Hammer ND, Cusumano CK, Hung CS, Chorell E, Aberg V, Walker JN, Seed PC, Almqvist F, et al. Small-molecule inhibitors target Escherichia coli amyloid biogenesis and biofilm formation. Nat Chem Biol 2009; 5:913-9; PMID:19915538; https://doi.org/10.1038/nchembio.242 19915538 PMC2838449

[170] Connolly KL, Roberts AL, Holder RC, Reid SD Dispersal of Group A streptococcal biofilms by the cysteine protease SpeB leads to increased disease severity in a murine model. PloS One 2011; 6:e18984; PMID:21547075; https://doi.org/10.1371/journal.pone.0018984 21547075 PMC3081844

[171] Park JH, Lee JH, Cho MH, Herzberg M, Lee J Acceleration of protease effect on Staphylococcus aureus biofilm dispersal. FEMS Microbiol Letters 2012; 335:31-8; PMID:22784033; https://doi.org/10.1111/j.1574-6968.2012.02635.x 22784033

[172] Yu C, Li X, Zhang N, Wen D, Liu C, Li Q Inhibition of biofilm formation by d-tyrosine: Effect of bacterial type and d-tyrosine concentration. Water Res 2016; 92:173-9; PMID:26854605; https://doi.org/10.1016/j.watres.2016.01.037 26854605

[173] Rumbo C, Vallejo JA, Cabral MP, Martinez-Guitian M, Perez A, Beceiro A, Bou G Assessment of antivirulence activity of several d-amino acids against Acinetobacter baumannii and Pseudomonas aeruginosa. J Antimicrobial Chemotherapy 2016; 71(12):3473-3481; PMID:27605598 27605598 10.1093/jac/dkw342

[174] Bhoopalan SV, Piekarowicz A, Lenz JD, Dillard JP, Stein DC nagZ Triggers Gonococcal Biofilm Disassembly. Scientific Reports 2016; 6:22372; PMID:26927542; https://doi.org/10.1038/srep22372 26927542 PMC4772129

[175] Nithyanand P, Beema Shafreen RM, Muthamil S, Karutha Pandian S Usnic acid inhibits biofilm formation and virulent morphological traits of Candida albicans. Microbiological Res 2015; 179:20-8; PMID:26411891; https://doi.org/10.1016/j.micres.2015.06.009 26411891

[176] Izadpanah A, Gallo RL Antimicrobial peptides. J Am Acad Dermatol 2005; 52:381-90; PMID:15761415; https://doi.org/10.1016/j.jaad.2004.08.026 15761415

[177] Li P, Wohland T, Ho B, Ding JL Perturbation of Lipopolysaccharide (LPS) Micelles by Sushi 3 (S3) antimicrobial peptide. The importance of an intermolecular disulfide bond in S3 dimer for binding, disruption, and neutralization of LPS. J Biol Chem 2004; 279:50150-6; PMID:15328339; https://doi.org/10.1074/jbc.M405606200 15328339

[178] Bhattacharjya S, Domadia PN, Bhunia A, Malladi S, David SA High-resolution solution structure of a designed peptide bound to lipopolysaccharide: transferred nuclear Overhauser effects, micelle selectivity, and anti-endotoxic activity. Biochemistry 2007; 46:5864-74; PMID:17469802; https://doi.org/10.1021/bi6025159 17469802

[179] Kharidia R, Liang JF The activity of a small lytic peptide PTP-7 on Staphylococcus aureus biofilms. J Microbiol 2011; 49:663-8; https://doi.org/10.1007/s12275-011-1013-5 21887652

[180] Mogi T, Kita K Gramicidin S and polymyxins: the revival of cationic cyclic peptide antibiotics. Cell Mol Life Sci 2009; 66:3821-6; PMID:19701717; https://doi.org/10.1007/s00018-009-0129-9 19701717 PMC11115702

[181] Ding JL, Li P, Ho B The Sushi peptides: structural characterization and mode of action against Gram-negative bacteria. Cell Mol Life Sci 2008; 65:1202-19; PMID:18213446; https://doi.org/10.1007/s00018-008-7456-0 18213446 PMC11131826

[182] Oren Z, Shai Y Mode of action of linear amphipathic alpha-helical antimicrobial peptides. Biopolymers 1998; 47:451-63; PMID:10333737; https://doi.org/10.1002/(SICI)1097-0282(1998)47:6%3c451::AID-BIP4%3e3.0.CO;2-F 10333737

[183] Mihajlovic M, Lazaridis T Antimicrobial peptides in toroidal and cylindrical pores. Biochim Et Biophysica Acta 2010; 1798:1485-93; PMID:20403332; https://doi.org/10.1016/j.bbamem.2010.04.004 20403332 PMC2885466

[184] Gottler LM, Ramamoorthy A Structure, membrane orientation, mechanism, and function of Pexiganan – A highly potent antimicrobial peptide designed from magainin. Biochim Et Biophysica Acta 2009; 1788:1680-6; PMID:19010301; https://doi.org/10.1016/j.bbamem.2008.10.009 19010301 PMC2726618

[185] Shai Y, Oren Z From “carpet” mechanism to de-novo designed diastereomeric cell-selective antimicrobial peptides. Peptides 2001; 22:1629-41; PMID:11587791; https://doi.org/10.1016/S0196-9781(01)00498-3 11587791

[186] Bierbaum G, Sahl HG Lantibiotics: mode of action, biosynthesis and bioengineering. Curr Pharmaceutical Biotechnol 2009; 10:2-18; PMID:19149587; https://doi.org/10.2174/138920109787048616 19149587

[187] Hasper HE, Kramer NE, Smith JL, Hillman JD, Zachariah C, Kuipers OP, de Kruijff B, Breukink E An alternative bactericidal mechanism of action for lantibiotic peptides that target lipid II. Science 2006; 313:1636-16377; PMID:16973881; https://doi.org/10.1126/science.1129818 16973881

[188] Hsu STD, Breukink E, Tischenko E, Lutters MAG, Kruijff B, Kaptein R, Bonvin AM, van Nuland NA The nisin-lipid II complex reveals a pyrophosphate cage that provides a blueprint for novel antibiotics. Nat Structural Mol Biol 2004; 11:963-7; https://doi.org/10.1038/nsmb830 15361862

[189] Parisot J, Carey S, Breukink E, Chan WC, Narbad A, Bonev B Molecular mechanism of target recognition by subtilin, a class I lanthionine antibiotic. Antimicrobial Agents Chemotherapy 2008; 52:612-8; PMID:17999970; https://doi.org/10.1128/AAC.00836-07 17999970 PMC2224776

[190] Saising J, Dube L, Ziebandt AK, Voravuthikunchai SP, Nega M, Gotz F Activity of gallidermin on Staphylococcus aureus and Staphylococcus epidermidis biofilms. Antimicrobial Agents Chemotherapy 2012; 56:5804-10; PMID:22926575; https://doi.org/10.1128/AAC.01296-12 22926575 PMC3486563

[191] Rienzo MAD, Banat IM, Dolman B, Winterburn J, Martin PJ Sophorolipid biosurfactants: Possible uses as antibacterial and antibiofilm agent. N Biotechnol 2015; 7:720-6. 10.1016/j.nbt.2015.02.009 25738966

[192] Incani V, Omar A, Prosperi-Porta G, Nadworny P Ag5IO6: novel antibiofilm activity of a silver compound with application to medical devices. Int J Antimicrobial Agents 2015; 45:586-93; PMID:25604278; https://doi.org/10.1016/j.ijantimicag.2014.09.008 25604278

[193] Percival SL, Finnegan S, Donelli G, Vuotto C, Rimmer S, Lipsky BA Antiseptics for treating infected wounds: Efficacy on biofilms and effect of pH. Critical Rev Microbiol 2014; 42(2):293–309; https://doi.org/10.3109/1040841X.2014.940495 25159044

[194] Kragol G, Hoffmann R, Chattergoon MA, Lovas S, Cudic M, Bulet P, Condie BA, Rosengren KJ, Montaner LJ, Otvos L Jr, et al. Identification of crucial residues for the antibacterial activity of the proline-rich peptide, pyrrhocoricin. Eur J Biochem / FEBS 2002; 269:4226-37; https://doi.org/10.1046/j.1432-1033.2002.03119.x 12199701

[195] Kragol G, Lovas S, Varadi G, Condie BA, Hoffmann R, Otvos L Jr The antibacterial peptide pyrrhocoricin inhibits the ATPase actions of DnaK and prevents chaperone-assisted protein folding. Biochemistry 2001; 40:3016-26; PMID:11258915; https://doi.org/10.1021/bi002656a 11258915

[196] Laszlo OJ, Insug O, Rogers ME, Consolvo PJ, Condie BA, Lovas S, Bulet P, Blaszczyk-Thurin M Interaction between heat shock proteins and antimicrobial peptides. Biochemistry 2000; 39:14150-9; PMID:11087363; https://doi.org/10.1021/bi0012843 11087363

[197] Gagnon MG, Roy RN, Lomakin IB, Florin T, Mankin AS, Steitz TA Structures of proline-rich peptides bound to the ribosome reveal a common mechanism of protein synthesis inhibition. Nucleic Acids Res 2016; 44:2439-50; PMID:26809677; https://doi.org/10.1093/nar/gkw018 26809677 PMC4797290

[198] Vizan JL, Hernandez-Chico C, del Castillo I, Moreno F The peptide antibiotic microcin B17 induces double-strand cleavage of DNA mediated by E. coli DNA gyrase. EMBO J 1991; 10:467-76. 1846808 10.1002/j.1460-2075.1991.tb07969.x PMC452668

[199] Finnegan S, Percival SL EDTA: An antimicrobial and antibiofilm agent for use in wound care. Adv Wound Care 2015; 4:415-21; PMID:26155384; https://doi.org/10.1089/wound.2014.0577 26155384 PMC4486448

[200] Zhang A, Mu H, Zhang W, Cui G, Zhu J, Duan J Chitosan coupling makes microbial biofilms susceptible to antibiotics. Scientific Reports 2013; 3:3364; PMID:24284335 24284335 10.1038/srep03364 PMC3842539

[201] Cho JH, Sung BH, Kim SC Buforins: histone H2A-derived antimicrobial peptides from toad stomach. Biochim Et Biophysica Acta 2009; 1788:1564-9; https://doi.org/10.1016/j.bbamem.2008.10.025 19041293

[202] Boman HG, Agerberth B, Boman A Mechanisms of action on Escherichia coli of cecropin P1 and PR-39, two antibacterial peptides from pig intestine. Infect Immunity 1993; 61:2978-84. 8514403 10.1128/iai.61.7.2978-2984.1993 PMC280948

[203] Subbalakshmi C, Sitaram N Mechanism of antimicrobial action of indolicidin. FEMS Microbiol Letters 1998; 160:91-6; PMID:9495018; https://doi.org/10.1111/j.1574-6968.1998.tb12896.x 9495018

[204] Hsu CH, Chen C, Jou ML, Lee AY, Lin YC, Yu YP, Huang WT, Wu SH Structural and DNA-binding studies on the bovine antimicrobial peptide, indolicidin: evidence for multiple conformations involved in binding to membranes and DNA. Nucleic Acids Res 2005; 33:4053-64; PMID:16034027; https://doi.org/10.1093/nar/gki725 16034027 PMC1179735

[205] Hell E, Giske CG, Nelson A, Römling U, Marchini G Human cathelicidin peptide LL37 inhibits both attachment capability and biofilm formation of Staphylococcus epidermidis. Lett Appl Microbiol 2010; 50:211-5; PMID:20002576; https://doi.org/10.1111/j.1472-765X.2009.02778.x 20002576

[206] Cirioni O, Giacometti A, Ghiselli R, Kamysz W, Orlando F, Mocchegiani F, Silvestri C, Licci A, Chiodi L, Lukasiak J, et al. Citropin 1.1-treated central venous catheters improve the efficacy of hydrophobic antibiotics in the treatment of experimental staphylococcal catheter-related infection. Peptides 2006; 27:1210-6; PMID:16289474; https://doi.org/10.1016/j.peptides.2005.10.007 16289474

[207] Willcox MD, Hume EB, Aliwarga Y, Kumar N, Cole N A novel cationic-peptide coating for the prevention of microbial colonization on contact lenses. J Appl Microbiol 2008; 105:1817-25; PMID:19016975; https://doi.org/10.1111/j.1365-2672.2008.03942.x 19016975

[208] Segev-Zarko L, Saar-Dover R, Brumfeld V, Mangoni ML, Shai Y Mechanisms of biofilm inhibition and degradation by antimicrobial peptides. Biochem J 2015; 468:259-70; PMID:25761937; https://doi.org/10.1042/BJ20141251 25761937

[209] Pimentel-Filho NJ, Martins MCF, Nogueira GB, Mantovani HC, Vanetti MCD Bovicin HC5 and nisin reduce Staphylococcus aureus adhesion to polystyrene and change the hydrophobicity profile and Gibbs free energy of adhesion. Int J Food Microbiol 2014; 190:1-8; PMID:25173449; https://doi.org/10.1016/j.ijfoodmicro.2014.08.004 25173449

[210] Konto-Ghiorghi Y, Mairey E, Mallet A, Dumenil G, Caliot E, Trieu-Cuot P, Dramsi S Dual role for pilus in adherence to epithelial cells and biofilm formation in Streptococcus agalactiae. PLoS Pathogens 2009; 5:e1000422; PMID:19424490; https://doi.org/10.1371/journal.ppat.1000422 19424490 PMC2674936

[211] Jacobsen SM, Stickler DJ, Mobley HL, Shirtliff ME Complicated catheter-associated urinary tract infections due to Escherichia coli and Proteus mirabilis. Clin Microbiol Rev 2008; 21:26-59; PMID:18202436; https://doi.org/10.1128/CMR.00019-07 18202436 PMC2223845

[212] Hung CS, Bouckaert J, Hung D, Pinkner J, Widberg C, DeFusco A, Auguste CG, Strouse R, Langermann S, Waksman G, et al. Structural basis of tropism of Escherichia coli to the bladder during urinary tract infection. Mol Microbiol 2002; 44:903-15; PMID:12010488; https://doi.org/10.1046/j.1365-2958.2002.02915.x 12010488

[213] Anderson GG, Palermo JJ, Schilling JD, Roth R, Heuser J, Hultgren SJ Intracellular bacterial biofilm-like pods in urinary tract infections. Science 2003; 301:105-7; PMID:12843396; https://doi.org/10.1126/science.1084550 12843396

[214] Justice SS, Hung C, Theriot JA, Fletcher DA, Anderson GG, Footer MJ, Hultgren SJ Differentiation and developmental pathways of uropathogenic Escherichia coli in urinary tract pathogenesis. Proc Natl Acad Sci U S A 2004; 101:1333-8; PMID:14739341; https://doi.org/10.1073/pnas.0308125100 14739341 PMC337053

[215] Wright KJ, Seed PC, Hultgren SJ Development of intracellular bacterial communities of uropathogenic Escherichia coli depends on type 1 pili. Cell Microbiol 2007; 9:2230-41; PMID:17490405; https://doi.org/10.1111/j.1462-5822.2007.00952.x 17490405

[216] Arslan SY, Leung KP, Wu CD The effect of lactoferrin on oral bacterial attachment. Oral Microbiol Immunol 2009; 24:411-6; PMID:19702956; https://doi.org/10.1111/j.1399-302X.2009.00537.x 19702956

[217] Wakabayashi H, Yamauchi K, Kobayashi T, Yaeshima T, Iwatsuki K, Yoshie H Inhibitory effects of lactoferrin on growth and biofilm formation of Porphyromonas gingivalis and Prevotella intermedia. Antimicrobial Agents Chemotherapy 2009; 53:3308-16; PMID:19451301; https://doi.org/10.1128/AAC.01688-08 19451301 PMC2715627

[218] Cusumano CK, Pinkner JS, Han Z, Greene SE, Ford BA, Crowley JR, Henderson JP, Janetka JW, Hultgren SJ Treatment and prevention of urinary tract infection with orally active FimH inhibitors. Sci Translational Med 2011; 3:109ra15; https://doi.org/10.1126/scitranslmed.3003021 PMC3694776 22089451

[219] Han Z, Pinkner JS, Ford B, Chorell E, Crowley JM, Cusumano CK, Campbell S, Henderson JP, Hultgren SJ, Janetka JW Lead optimization studies on FimH antagonists: discovery of potent and orally bioavailable ortho-substituted biphenyl mannosides. J Med Chem 2012; 55:3945-59; PMID:22449031; https://doi.org/10.1021/jm300165m 22449031 PMC3375398

[220] Han Z, Pinkner JS, Ford B, Obermann R, Nolan W, Wildman SA, Hobbs D, Ellenberger T, Cusumano CK, Hultgren SJ, et al. Structure-based drug design and optimization of mannoside bacterial FimH antagonists. J Med Chem 2010; 53:4779-92; PMID:20507142; https://doi.org/10.1021/jm100438s 20507142 PMC2894565

[221] Guiton PS, Cusumano CK, Kline KA, Dodson KW, Han Z, Janetka JW, Henderson JP, Caparon MG, Hultgren SJ Combinatorial small-molecule therapy prevents uropathogenic Escherichia coli catheter-associated urinary tract infections in mice. Antimicrobial Agents Chemotherapy 2012; 56:4738-45; PMID:22733070; https://doi.org/10.1128/AAC.00447-12 22733070 PMC3421856

[222] Greene SE, Pinkner JS, Chorell E, Dodson KW, Shaffer CL, Conover MS, Livny J, Hadjifrangiskou M, Almqvist F, Hultgren SJ Pilicide ec240 disrupts virulence circuits in uropathogenic Escherichia coli. MBio 2014; 5:e02038; PMID:25352623; https://doi.org/10.1128/mBio.02038-14 25352623 PMC4217179

[223] Siddiq DM, Darouiche RO New strategies to prevent catheter-associated urinary tract infections. Nat Rev Urol 2012; 9:305-14; PMID:22508462; https://doi.org/10.1038/nrurol.2012.68 22508462

[224] Jiang P, Li J, Han F, Duan G, Lu X, Gu Y, Yu W Antibiofilm activity of an exopolysaccharide from marine bacterium Vibrio sp. QY101. PloS One 2011; 6:e18514; PMID:21490923; https://doi.org/10.1371/journal.pone.0018514 21490923 PMC3072402

[225] Rendueles O, Kaplan JB, Ghigo JM Antibiofilm polysaccharides. Environmental Microbiol 2013; 15:334-46; PMID:22730907; https://doi.org/10.1111/j.1462-2920.2012.02810.x 22730907 PMC3502681

[226] Das T, Manefield M Pyocyanin promotes extracellular DNA release in Pseudomonas aeruginosa. PloS One 2012; 7:e46718; PMID:23056420; https://doi.org/10.1371/journal.pone.0046718 23056420 PMC3466280

[227] Wu S, Liu G, Jin W, Xiu P, Sun C Antibiofilm and Anti-Infection of a Marine Bacterial Exopolysaccharide Against Pseudomonas aeruginosa. Front Microbiol 2016; 7:102; PMID:26903981 26903981 10.3389/fmicb.2016.00102 PMC4744842

[228] Pihl M, JR D, CdP LE, G S Differential effects of Pseudomonas aeruginosa on biofilm formation by different strains of Staphylococcus epidermidis. FEMS Immunol Med Microbiol 2010; 59:439-46; PMID:20528934; https://doi.org/10.1111/j.1574-695X.2010.00697.x 20528934

[229] Qin Z, Yang L, Qu D, Molin S, Tolker-Nielsen T Pseudomonas aeruginosa extracellular products inhibit staphylococcal growth, and disrupt established biofilms produced by Staphylococcus epidermidis. Microbiology 2009; 155:2148-56; PMID:19389780; https://doi.org/10.1099/mic.0.028001-0 19389780

[230] Bendaoud M, Vinogradov E, Balashova NV, Kadouri DE, Kachlany SC, Kaplan JB Broad-spectrum biofilm inhibition by Kingella kingae exopolysaccharide. J Bacteriol 2011; 193:3879-86; PMID:21602333; https://doi.org/10.1128/JB.00311-11 21602333 PMC3147541

[231] Valle J, Da Re S, Henry N, Fontaine T, Balestrino D, Latour-Lambert P, Ghigo JM Broad-spectrum biofilm inhibition by a secreted bacterial polysaccharide. Proc Natl Acad Sci U S A 2006; 103:12558-63; PMID:16894146; https://doi.org/10.1073/pnas.0605399103 16894146 PMC1567917

[232] Rendueles O, Travier L, Latour-Lambert P, Fontaine T, Magnus J, Denamur E, Ghigo JM Screening of Escherichia coli species biodiversity reveals new biofilm-associated anti-adhesion polysaccharides. mBio 2011; 2:e00043-e11; PMID:21558434; https://doi.org/10.1128/mBio.00043-11 21558434 PMC3101779

[233] Sayem SM, Manzo E, Ciavatta L, Tramice A, Cordone A, Zanfardino A, De Felice M, Varcamonti M Anti-biofilm activity of an exopolysaccharide from a sponge-associated strain of Bacillus licheniformis. Microbial Cell Factories 2011; 10:74; PMID:21951859; https://doi.org/10.1186/1475-2859-10-74 21951859 PMC3196911

[234] Kim Y, Oh S, Kim SH Released exopolysaccharide (r-EPS) produced from probiotic bacteria reduce biofilm formation of enterohemorrhagic Escherichia coli O157:H7. Biochem Biophys Res Commun 2009; 379:324-9; PMID:19103165; https://doi.org/10.1016/j.bbrc.2008.12.053 19103165

[235] Yu S, Su T, Wu H, Liu S, Wang D, Zhao T, Jin Z, Du W, Zhu MJ, Chua SL, et al. PslG, a self-produced glycosyl hydrolase, triggers biofilm disassembly by disrupting exopolysaccharide matrix. Cell Res 2015; 25:1352-67; PMID:26611635; https://doi.org/10.1038/cr.2015.129 26611635 PMC4670989

[236] Romling U, Galperin MY, Gomelsky M Cyclic di-GMP: the first 25 years of a universal bacterial second messenger. Microbiol Mol Biol Rev 2013; 77:1-52; PMID:23471616; https://doi.org/10.1128/MMBR.00043-12 23471616 PMC3591986

[237] Chua SL, Liu Y, Yam JK, Chen Y, Vejborg RM, Tan BG, Kjelleberg S, Tolker-Nielsen T, Givskov M, Yang L Dispersed cells represent a distinct stage in the transition from bacterial biofilm to planktonic lifestyles. Nat Commun 2014; 5:4462; PMID:25042103; https://doi.org/10.1038/ncomms5462 25042103

[238] Sambanthamoorthy K, Luo C, Pattabiraman N Identification of small molecules inhibiting diguanylate cyclases to control bacterial biofilm development. Biofouling 2014; 30:17-28; PMID:24117391; https://doi.org/10.1080/08927014.2013.832224 24117391 PMC4120261

[239] Lieberman OJ, Orr MW, Wang Y, Lee VT High-throughput screening using the differential radial capillary action of ligand assay identifies ebselen as an inhibitor of diguanylate cyclases. ACS Chem Biol 2014; 9:183-92; PMID:24134695; https://doi.org/10.1021/cb400485k 24134695 PMC4545405

[240] Mueller RS, Beyhan S, Saini SG, Yildiz FH, Bartlett DH Indole acts as an extracellular cue regulating gene expression in Vibrio cholerae. J Bacteriol 2009; 191:3504-16; PMID:19329638; https://doi.org/10.1128/JB.01240-08 19329638 PMC2681914

[241] Lee J, Page R, Garcia-Contreras R, Palermino JM, Zhang XS, Doshi O, et al. Structure and function of the Escherichia coli protein YmgB: a protein critical for biofilm formation and acid-resistance. J Mol Biol 2007; 373:11-26; PMID:17765265; https://doi.org/10.1016/j.jmb.2007.07.037 17765265 PMC2185545

[242] Lee J, Jayaraman A, Wood TK Indole is an inter-species biofilm signal mediated by SdiA. BMC Microbiol 2007; 7:42 17511876 10.1186/1471-2180-7-42 PMC1899176

[243] Nishino K, Nikaido E, Yamaguchi A Regulation of multidrug efflux systems involved in multidrug and metal resistance of Salmonella enterica serovar Typhimurium. J Bacteriol 2007; 189:9066-75; PMID:17933888; https://doi.org/10.1128/JB.01045-07 17933888 PMC2168627

[244] Bunders CA, Minvielle MJ, Worthington RJ, Ortiz M, Cavanagh J, Melander C Intercepting bacterial indole signaling with flustramine derivatives. J Am Chem Society 2011; 133:20160-3; PMID:22091927; https://doi.org/10.1021/ja209836z 22091927 PMC3246311

[245] Durig A, Kouskoumvekaki I, Vejborg RM, Klemm P Chemoinformatics-assisted development of new anti-biofilm compounds. Applied Microbiol Biotechnol 2010; 87:309-17; PMID:20204615; https://doi.org/10.1007/s00253-010-2471-0 20204615

[246] Amalaradjou MAR, Venkitanarayanan K Antibiofilm effect of Octenidine Hydrochloride on Staphylococcus aureus, MRSA and VRSA. Pathogens 2014; 3:404-16; PMID:25437807; https://doi.org/10.3390/pathogens3020404 25437807 PMC4243453

[247] Hirsch T, Jacobsen F, Rittig A A comparative in vitro study of cell toxicity of clinically used antiseptics. Hautarzt 2009; 60:984-91; PMID:19812986; https://doi.org/10.1007/s00105-009-1842-x 19812986

[248] Gopal R, Kim YG, Lee JH, Lee SK, Chae JD, Son BK, Seo CH, Park Y Synergistic effects and antibiofilm properties of chimeric peptides against multidrug-resistant acinetobacter baumannii strains. Antimicrobial Agents Chemotherapy 2014; 58:1622-9; PMID:24366740; https://doi.org/10.1128/AAC.02473-13 24366740 PMC3957903

[249] Cady NC, McKean KA, Behnke J, Kubec R, Mosier AP, Kasper SH, Burz DS, Musah RA Inhibition of biofilm formation, quorum sensing and infection in Pseudomonas aeruginosa by natural products-inspired organosulfur compounds. PloS One 2012; 7:e38492; PMID:22715388; https://doi.org/10.1371/journal.pone.0038492 22715388 PMC3371053

[250] Betzig E, Patterson GH, Sougrat R, Lindwasser OW, Olenych S, Bonifacino JS, Davidson MW, Lippincott-Schwartz J, Hess HF Imaging intracellular fluorescent proteins at nanometer resolution. Science 2006; 313:1642-5; PMID:16902090; https://doi.org/10.1126/science.1127344 16902090

[251] Hess ST, Girirajan TP, Mason MD Ultra-high resolution imaging by fluorescence photoactivation localization microscopy. Biophysical J 2006; 91:4258-72; PMID:16980368; https://doi.org/10.1529/biophysj.106.091116 16980368 PMC1635685

[252] Rust MJ, Bates M, Zhuang X Subdiffraction-limit imaging by stochastic optical reconstruction microscopy (STORM). Nat Methods 2006; 3:793-5; PMID:16896339; https://doi.org/10.1038/nmeth929 16896339 PMC2700296

[253] Abed SE, Ibnsouda SK, Latrache H, Hamadi F Scanning Electron Microscopy (SEM) and Environmental SEM: Suitable tools for study of adhesion stage and biofilm formation. In Scanning Electron Microscopy, Kazmiruk V., ed. Rijeka, Croatia: InTech 2012.

[254] Vidigal PG, Musken M, Becker KA, Haussler S, Wingender J, Steinmann E, Kehrmann J, Gulbins E, Buer J, Rath PM, et al. Effects of green tea compound epigallocatechin-3 gallate against Stenotrophomonas maltophilia infection and biofilm. PloS One 2014; 9:e92876; PMID:24690894; https://doi.org/10.1371/journal.pone.0092876 24690894 PMC3972220

[255] Magesh H, Kumar A, Alam A, Sekar U, Sumantran VN, Vaidyanathan R Identification of natural compounds which inhibit biofilm formation in clinical isolates of Klebsiella pneumoniae. Indian J Exp Biol 2013; 51:764-72; PMID:24377137 24377137

[256] Gopu V, Meena CK, Shetty PH Quercetin influences quorum sensing in food borne bacteria: In-vitro and in-silico evidence. PloS One 2015; 10:e0134684; PMID:26248208; https://doi.org/10.1371/journal.pone.0134684 26248208 PMC4527846

[257] Adil M, Singh K, Verma PK, Khan AU Eugenol-induced suppression of biofilm-forming genes in Streptococcus mutans: An approach to inhibit biofilms. J Global Antimicrobial Resistance 2014; 2:286-92; PMID:27873689; https://doi.org/10.1016/j.jgar.2014.05.006 27873689

[258] Liang ZX The expanding roles of c-di-GMP in the biosynthesis of exopolysaccharides and secondary metabolites. Natural Product Reports 2015; 32:663-83; PMID:25666534; https://doi.org/10.1039/C4NP00086B 25666534

[259] Park SC, Park Y, Hahm KS The role of antimicrobial peptides in preventing multidrug-resistant bacterial infections and biofilm formation. Int J Mol Sci 2011; 12:5971-92; PMID:22016639; https://doi.org/10.3390/ijms12095971 22016639 PMC3189763

[260] Shadia MAA, Aeron A Bacterial Biofilm: Dispersal and Inhibition Strategies. SAJ Biotechnol 2014; 1:105.

[261] Vizan JL, Hernandez-Chico C, Castillo I, Moreno F The Antibiotic Microcin B17 is a DNA Gyrase Poison:Characterisation of the mode of inhibition. EMBO J 1991; 10:467-76; PMID:1846808 1846808 10.1002/j.1460-2075.1991.tb07969.x PMC452668

[262] Kim JY, Park SC, Yoon MY, Hahm KS, Park Y C-terminal amidation of PMAP-23: translocation to the inner membrane of Gram-negative bacteria. Amino Acids 2011; 40:183-95; PMID:20512598; https://doi.org/10.1007/s00726-010-0632-1 20512598

[263] Sae-tan S, Grove KA, Kennett MJ, Lambert JD (−)-Epigallocatechin-3 gallate increases the expression of genes related to fat oxidation in the skeletal muscle of high fat-fed mice. Food Funct 2011; 2:111-6; PMID:21779555; https://doi.org/10.1039/c0fo00155d 21779555 PMC3400462

[264] de Manincor M The reason why mango should be in everyone's diet. flipper e nuvola 2013 http://flipper.diff.org/apptagsaccount/items/5356 (accessed 4 18, 2017).

[265] Shiner EK, Rumbaugh KP, Williams SC Interkingdom signaling: Deciphering the language of acyl homoserine lactones. FEMS Microbiol Rev 2005; 29:935-47; PMID:16219513; https://doi.org/10.1016/j.femsre.2005.03.001 16219513

[266] National Center for Biotechnology Information PubChem Compound Database; CID=5770, 2006 https://pubchem.ncbi.nlm.nih.gov/compound/5770 (accessed April 18, 2017).

[267] National Center for Biotechnology Information PubChem Compound Database; CID=969516, 2016 https://pubchem.ncbi.nlm.nih.gov/compound/969516 (accessed April 18, 2017).

[268] Soh PN, Witkowski B, Olagnier D, Nicolau ML, Garcia-Alvarez MC, Berry A, Benoit-Vical F In vitro and in vivo properties of ellagic acid in malaria treatment. Antimicrobial Agents Chemotherapy 2009; 53:1100-6; PMID:19015354; https://doi.org/10.1128/AAC.01175-08 19015354 PMC2650562

[269] National Center for Biotechnology Information PubChem Compound Database; CID=16129778, 2014 https://pubchem.ncbi.nlm.nih.gov/compound/16129778 (accessed April 18, 2017).

[270] National Center for Biotechnology Information PubChem Compound Database; CID=3314, 2004 https://pubchem.ncbi.nlm.nih.gov/compound/3314 (accessed April 18, 2017).

[271] National Center for Biotechnology Information PubChem Compound Database; CID=2353, 2004 https://pubchem.ncbi.nlm.nih.gov/compound/2353 (accessed April 18, 2017).

[272] National Center for Biotechnology Information PubChem Compound Database; CID=5646, 2005 https://pubchem.ncbi.nlm.nih.gov/compound/5646 (accessed April 18, 2017).

[273] National Center for Biotechnology Information PubChem Compound Database; CID=5311054, 2005 https://pubchem.ncbi.nlm.nih.gov/compound/5311054 (accessed April 18, 2017).

[274] Ejim L, Farha MA, Falconer SB, Wildenhain J, Coombes BK, Tyers M, Brown ED, Wright GD Combinations of antibiotics and nonantibiotic drugs enhance antimicrobial efficacy. Nat Chem Biol 2011; 7:348-50; PMID:21516114; https://doi.org/10.1038/nchembio.559 21516114

[275] Yamamura H, Suzuki K, Uchibori K, Miyagawa A, Kawai M, Ohmizo C, Katsu T Mimicking an antimicrobial peptide polymyxin B by use of cyclodextrin. Royal Society Chem 2012; 48:892-4. 10.1039/c1cc16369h 22143262

[276] National Center for Biotechnology Information PubChem Compound Database; CID=73357, 2005 https://pubchem.ncbi.nlm.nih.gov/compound/73357 (accessed April 18, 2017).

[277] National Center for Biotechnology Information PubChem Compound Database; CID=16219761, 2007 https://pubchem.ncbi.nlm.nih.gov/compound/16219761 (accessed April 18, 2017).

[278] Scaffaroa R, Bottaa L, Gallo G Photo-oxidative degradation of poly (ethylene-co-vinyl acetate)/nisin antimicrobial films. Polymer Degradation Stability 2012; 97:653-60; https://doi.org/10.1016/j.polymdegradstab.2012.01.003

[279] National Center for Biotechnology Information PubChem Compound Database; CID=16130064, 2007 https://pubchem.ncbi.nlm.nih.gov/compound/16130064 (accessed April 18, 2017).

[280] National Center for Biotechnology Information PubChem Compound Database; CID=16132391, 2007 https://pubchem.ncbi.nlm.nih.gov/compound/16132391 (accessed April 18, 2017).

[281] National Center for Biotechnology Information PubChem Compound Database; CID=73348284, 2014 https://pubchem.ncbi.nlm.nih.gov/compound/73348284 (accessed April 18, 2017).

[282] National Center for Biotechnology Information PubChem Compound Database; CID=9552079, 2006 https://pubchem.ncbi.nlm.nih.gov/compound/9552079 (accessed April 18, 2017).

[283] Baccile N, Pedersen JS, Pehau-Arnaudet G, Van Bogaert INA Surface charge of acidic sophorolipid micelles: effect of base and time. Royal Society Chem 2013; 9:4911-22.

[284] National Center for Biotechnology Information PubChem Compound Database; CID=20977, 2005 https://pubchem.ncbi.nlm.nih.gov/compound/20977 (accessed April 18, 2017).

[285] National Center for Biotechnology Information PubChem Compound Database; CID=101097383, 2015 https://pubchem.ncbi.nlm.nih.gov/compound/101097383 (accessed April 18, 2017).

[286] Collin F, Karkare S, Maxwell A Exploiting bacterial DNA gyrase as a drug target: current state and perspectives. Appl Microbiol Biotechnol 2011; 92:479-97; PMID:21904817; https://doi.org/10.1007/s00253-011-3557-z. 21904817 PMC3189412

[287] National Center for Biotechnology Information PubChem Compound Database; CID=16131340, 2007 https://pubchem.ncbi.nlm.nih.gov/compound/16131340 (accessed April 18, 2017).

[288] National Center for Biotechnology Information PubChem Compound Database; CID=6224, 2009 https://pubchem.ncbi.nlm.nih.gov/compound/6224 (accessed April 18, 2017).

[289] National Center for Biotechnology Information PubChem Compound Database; CID=6144, 2007 https://pubchem.ncbi.nlm.nih.gov/compound/6144 (accessed April 18, 2017).

[290] Kota S, Adibhatla KSBR, Venkaiah CN Improved process for the preparation of cadexomer iodine. Natco Pharma Limited. Patent no. WO2008117300 A2; 2008.

[291] National Center for Biotechnology Information PubChem Compound Database; CID=16198951, 2007 https://pubchem.ncbi.nlm.nih.gov/compound/16198951 (accessed April 18, 2017).

[292] Franklin M, Nivens D, Weadge J, Howell P Biosynthesis of the Pseudomonas aeruginosa Extracellular Polysaccharides, Alginate, Pel, and Psl. Frontiers Microbiol 2011; 2(167); PMCID: ; https://doi.org/10.3389/fmicb.2011.00167 21991261 PMC3159412

[293] Mann EE, Wozniak DJ Pseudomonas biofilm matrix composition and niche biology. FEMS Microbiol Rev 2012; 36:893-916; PMID:22212072; https://doi.org/10.1111/j.1574-6976.2011.00322.x 22212072 PMC4409827

[294] Jennings LK, Storek KM, Ledvina HE, Coulon C, Marmont LS, Sadovskaya I, Secor PR, Tseng BS, Scian M, Filloux A, et al. Pel is a cationic exopolysaccharide that cross-links extracellular DNA in the Pseudomonas aeruginosa biofilm matrix. Proc Natl Acad Sci US A 2015; 112:11353-8; PMID:26311845; https://doi.org/10.1073/pnas.1503058112 26311845 PMC4568648

[295] Mistretta N, Danve E, Moreau M Conjugates obtained by reductive amination of the pneumococcus serotype 5 capsular polysaccharide. Aventis Pasteur S.A. Patent no. US7812006 B2; 2010.

[296] Kim JS, Ha TY, Ahn J, Kim S Analysis and distribution of esculetin in plasma and tissues of rats after oral administration. Preventive Nutrition Food Sci 2014; 19:321-6; PMID:25580397; https://doi.org/10.3746/pnf.2014.19.4.321 25580397 PMC4287325

[297] Maher P, Akaishi T, Abe K Flavonoid fisetin promotes ERK-dependent long-term potentiation and enhances memory. Proc Natl Acad Sci U S A 2006; 103:16568-73; PMID:17050681; https://doi.org/10.1073/pnas.0607822103 17050681 PMC1637622

[298] National Center for Biotechnology Information PubChem Compound Database; CID=51166, 2005 https://pubchem.ncbi.nlm.nih.gov/compound/51166 (accessed April 18, 2017).

